# Indecision and delays are the parents of failure—taming them algorithmically by synthesizing delay-resilient control

**DOI:** 10.1007/s00236-020-00374-7

**Published:** 2020-02-20

**Authors:** Mingshuai Chen, Martin Fränzle, Yangjia Li, Peter N. Mosaad, Naijun Zhan

**Affiliations:** 1 Lehrstuhl für Informatik 2, RWTH Aachen University, Aachen, Germany; 2 Department of Computing Science, Carl von Ossietzky Universität Oldenburg, Oldenburg, Germany; 3 University of Tartu, Tartu, Estonia; 4 State Key Laboratory of Computer Science, Institute of Software, Chinese Academy of Sciences, Beijing, China; 5 University of Chinese Academy of Sciences, Beijing, China

## Abstract

The possible interactions between a controller and its environment can naturally be modelled as the arena of a two-player game, and adding an appropriate winning condition permits to specify desirable behavior. The classical model here is the positional game, where both players can (fully or partially) observe the current position in the game graph, which in turn is indicative of their mutual current states. In practice, neither sensing and actuating the environment through physical devices nor data forwarding to and from the controller and signal processing in the controller are instantaneous. The resultant delays force the controller to draw decisions before being aware of the recent history of a play and to submit these decisions well before they can take effect asynchronously. It is known that existence of a winning strategy for the controller in games with such delays is decidable over finite game graphs and with respect to $\omega$-regular objectives. The underlying reduction, however, is impractical for non-trivial delays as it incurs a blow-up of the game graph which is exponential in the magnitude of the delay. For safety objectives, we propose a more practical incremental algorithm successively synthesizing a series of controllers handling increasing delays and reducing the game-graph size in between. It is demonstrated using benchmark examples that even a simplistic explicit-state implementation of this algorithm outperforms state-of-the-art symbolic synthesis algorithms as soon as non-trivial delays have to be handled. We furthermore address the practically relevant cases of non-order-preserving delays and bounded message loss, as arising in actual networked control, thereby considerably extending the scope of regular game theory under delay.

## Introduction

Two-player games played over game arenas are established models for the synthesis of correct-by-construction reactive controllers [10, 23]. A finite game graph is used to formalize the possible actions of the players; it is complemented by a winning condition specifying desirable properties of infinite paths by means of an acceptance condition or a specification in temporal logic. Frequently, the game is played on a finite graph alternating moves by two players; the first player is the controller (sometimes called “ego” player) and the second player is its environment (“alter”), which may be uncooperative, erratic, or even malicious. Correct controllers thus have to be able to counteract any environmental actions, i.e., they need a sure winning strategy in the game. Controller synthesis can thus be understood as search for a winning strategy for ego. In this paper, we are interested in the synthesis problem when the interaction of a controller and its environment is described by a safety game [23], i.e., an infinite two-player game on finite graphs comprising “unsafe” states that the controller should avoid visiting. The controller consequently loses whenever the play eventually reaches such an unsafe state.

These safety games have traditionally been investigated in a setting where the current position in the game is either fully known (“perfect information”) or known up to certain observability constraints (“imperfect/incomplete information”). In this article, we address the problem of control under delays in perception and action. This can be understood as a form of imperfect information, as decisions by the controller have to be drawn based on delayed state observation—i.e., with the recent game history being opaque to the controller—and in advance—i.e., well before the actual situation where the action takes effect is fully determined. Such games have numerous practical applications, especially in networked control settings like cooperative driving, where observation of and influence on other cars’ states are delayed by communication protocols severely restricting frequency of certain message types in order to keep overall channel usage sustainable under the pertinent severe bandwidth constraints. Other sources of delay can be found in complex signal processing chains, for example when interpretation of the environmental situation is based on computer vision.

It is intuitively obvious that such delay renders control harder: the controller has to decide in advance and based on dated information. Already at the time of decision, this dated information may no longer be fully indicative of the current situation due to the interim development, and it will even less provide a precise prediction of the expected situation at the time of the decision taking effect in the environment. The existence of a winning strategy for the controller under such delays nevertheless is decidable over finite game graphs and with respect to $\omega$-regular objectives. A straightforward reduction to delay-free games based on a product construction is impractical for non-trivial delays as it incurs a blow-up of the game graph which is strictly exponential in the magnitude of the delay, as observed by Tripakis [30].

In this article, we follow Tripakis’ quest for more efficient algorithms. For safety objectives, we therefore expose a more practical incremental algorithm synthesizing a series of controllers handling increasing delays and reducing game-graph size in between. We demonstrate on benchmark examples that even a simplistic explicit-state implementation of this algorithm outperforms state-of-the-art symbolic synthesis algorithms as soon as non-trivial delays have to be handled. Beyond the case of message- and order-preserving, sequential delay, we furthermore address the practically relevant cases of non-order-preserving delays as well as of bounded message loss, as arising in actual networked control. We thereby considerably extend the scope of regular game theory under delay as explained below.

*Related work.* In the literature on games and reactive synthesis, constraints on observation and interaction are reflected by corresponding restrictions on the information frames available to the players. The majority of the results about two-player games adopt the hypothesis of *perfect information* (or *synchronous behavior* in reactive synthesis), where fixed-point algorithms for the computation of winning strategies exist [7, 8, 10]. In this case, the controller is aware of the exact current (and past) state of its environment when selecting its next control action. In practice, the hypothesis of perfect state information is often not justified due to only part of the environment state being observable by the controller. Reif [26] has consequently studied games of *incomplete information* and Pnueli and Rosner [25] have considered the synthesis of a reactive *asynchronous* module, where the program to be synthesized may observe only a subsequence of a play in the game (though not over an explicit game graph). It is known that this approach can handle linear temporal logic (LTL) synthesis with incomplete information. Essentially, non-determinism of the automata can be used to guess the missing information, guaranteeing that no guess violates the specification [31]. Alternative methods have been proposed thereafter for asynchronous synthesis, see e.g. [2, 21, 27]. An orthogonal direction has been exploited by Kupferman and Vardi in [22] to consider the problem of synthesis with incomplete information for branching-time logics captured by computational tree logic (CTL) and $\hbox {CTL}^*$. In their setting, the current position in the game is known up to certain observability constraints, that is, the state space of the game graph is partitioned into finite observations while the controller cannot distinguish states pertaining to the same observation. Such of setting of partial observability has been recently carried further to the synthesis of $\hbox {LTL}_f$ (LTL over finite traces) and $\hbox {LDL}_f$ (linear dynamic logic over finite traces) [12]. Similarly, Wulf, Doyen, and Raskin in [32] and Chatterjee, Doyen, Henzinger, and Raskin in [10] study two-player games on graphs with $\omega$-regular objectives subject to partial observability of the current (and past) game state. Recent state information however is well available in these settings, as no restriction concerning the minimum age of observable state information is imposed. As the latter is an increasingly relevant problem in, e.g., networked control with its non-trivial end-to-end communication latencies, we here address the problem of two-player safety games played over an explicit game arena subject to *delayed observation* and *delayed action* of the controlled process, obtaining a specific and practically extremely relevant case of imperfect information amenable to optimized synthesis algorithms.

A seminal theory of delayed $\omega$-regular games dates back to the 1970s, shortly after the seminal Büchi–Landweber theorem [9], when Hosch and Landweber [17] investigated constant delay strategies in regular games and exploited a result of Even and Meyer [13] from Boolean circuit theory to establish a bound for delays. The problem was then revisited by Holtmann, Kaiser, and Thomas [15, 16] and more recently by Klein and Zimmermann [19, 20, 33], leading to the first comprehensive study with a flurry of new theoretical results concerning, e.g., the complexity of solving such games and the necessary and sufficient constant delay. The setting underlying these notions of delayed games, however, is complementary to ours, as they are rooted in the setting of Gale-Stewart games [14], where an input player chooses an input sequence and an output player tries to add a corresponding output sequence such that the combined sequence satisfies a specification. In the Gale-Stewart setting, turn-based playing means that the output player has to issue the *i*th output letter in immediate response to the first *i* input letters, while a delay according to [13, 15–17, 19, 20, 33] implies that the delayed output player is allowed to lag behind and issue the *i*th output letter after seeing more than just *i* input letters. In detail, there is a function $f:{\mathbb {N}}\rightarrow {\mathbb {N}}$ such that the output player has to produce the *i*th letter of the output string only when $i + \sum _{j=0}^i f(j)$ letters of the input string are available. Thus, delay comes as an advantage to the output player, as the input player actually grants the output player a lookahead; the burden for the output player is just that she may have to memorize (a finite abstraction of) infinite lookahead if delay is unbounded in that $\sum _{j=0}^i f(j)$ diverges. As delay is an advantage in this setting, questions like “what delay suffices for winning the game if it is winnable at all” make sense and have been answered by proving exponential bounds in the size of the state-space of the $\omega$-regular specification automaton.

In the setting of such Gale-Stewart games, our notion of reactive control based on delayed state information and thus imperfect information coincides to the notion of “shift” introduced by Büchi and Landweber in [8] rather than to the notion of “delay”. While this may provoke the conception that our setting can thus be understood as asking for a strategy of the input player—whose strategic strength suffers from having to grant that lookahead—rather than for the output player in a delayed Gale-Stewart game, this is not the case. The notions of “delay” and “shift” are unfortunately not duals that could be transformed into each other by exchanging the roles of the input and the output player. Just like concurrent games [15], and in contrast to Gale-Stewart games under delay, games under shift are not even determined, as the following example shows: consider the Gale-Stewart game where both players play actions from alphabet $\{0,1\}$ and the output player wins whenever it in turn *i* issues the very same letter as the input player submits in the same turn *i*. Clearly, the output player here has a winning strategy when there is neither shift nor delay, but with a shift of 1—i.e., if the output player has to submit its *i*th letter of the input based on knowing the input up to letter $i-1$—neither the input nor the output player have a winning strategy.

For the simplest setting of our notion of delay, namely that the delay between in- and output is constant, we can nevertheless exploit a similar reduction to games of perfect information as the oblivious-delay construction of Zimmermann [33], which in the case of constant delay exploits a product construction on the game graph essentially representing a synchronous concurrent composition of the graph with a shift register implementing the delays. As in the Gale-Stewart setting, such a reduction proves that strategy synthesis procedures for games without delay do in principle suffice for solving games with finite or constantly bounded delay. As this comes at the price of an exponential blow-up in the game graph (for our $\omega$-regular game-graph setting) or specification automaton (for the Gale-Stewart setting), we share with [13, 15–17, 19, 20, 33] the objective of finding more efficient strategy synthesis methods. Due to the reversed roles of delay or rather shift, the lines of work do, however, depart from here: while the Gale-Stewart-based approach aims to minimize the necessary delay of the output player and the amount of memory that the output player additionally has to manipulate in order to ensure a win, we in contrast essentially ask for maximizing the shift under which the input player can still enforce a win against the output player and for efficiently constructing a delay-resilient winning strategy for the input player. As delay and shift are not duals that could be transformed into each other by exchanging the roles of input and output player, the resulting constructions are substantially different.

To this setting of constant delay, we add two practically relevant notions of delay that are not covered by the setting of delay in [13, 15–17, 19, 20, 33] and its counterpart “shift”, namely out-of-order delivery of messages and message loss (see Sect. 4). Notions of out-of-order delivery of messages and of message loss are natural in embedded control, but would be awkward to describe in the Gale-Stewart setting, as they would there amount to the output player having to select her next action based on a permutation (out-of-order) of a subsequence (message loss) of a prefix (delay, a.k.a. shift) of the outputs. It therefore comes as no surprise that these in practice frequently arising variants of imperfect delayed state information have not yet been addressed in the Gale-Stewart setting.

It is finally worth noting that the notion of delay employed in this paper as well as the related notion in the above Gale-Stewart setting are substantially different from delays in timed games and their synthesis algorithms, like Uppaal-Tiga [3], as well as from the notion of delay used in the discrete-event system community, e.g. [1, 24]. In timed games, delay refers to the possibility to deliberately protract the next control action, i.e., a single event. Up-to-date positional information, however, is always fully transparent to both players in timed games. In our setting, delay refers to a time lag imposed when obtaining positional information, modelling the end-to-end latency of information distribution in a communication network. Up-to-date positional information thus is opaque to the players as long as it resides in a queue modelling the network, where state information as well as control events of multiple different ages coexist and pipeline towards delivery. Such pipelining of control actions is lacking in the models of delay from [3] or [24], where only one controllable event can be latent at any time and just the time of its actual execution is determined by the controller or the environment, respectively. Meanwhile, the model of delay in [1] is different from ours as it leads to non-regular languages.

## Safety games under delayed information

Given a set *A*, we denote its powerset by $2^A$, the set of finite sequences over *A* by $A^*$, and the set of infinite sequences over *A* by $A^\omega$. The relative complement of a set *B* in *A* is denoted by $A {\setminus } B = \{x \in A \mid x \not \in B \}$. An empty sequence is denoted by $\varepsilon$, and ${\mathbb {N}}$ is the set of non-negative integers.

### Games with perfect information

The plays we consider are played on finite bipartite game graphs as known from $\omega$-regular games, see e.g. [29]:

#### Definition 1

*(Two-player game graph)* A *finite game graph* is of the form $G = \langle S, s_0, S_0, S_1, \varSigma _0, \varSigma _1, \rightarrow \rangle$, where *S* is a finite (non-empty) set of states and $S_0\subset S$ and $S_1\subset S$ define a partition of *S*, with $S_i$ containing the states where it is the turn of player *i* to perform an action. $s_0 \in S_0$ is the *initial* state. $\varSigma _0$ and $\varSigma _1$ are disjoint finite alphabets of *actions* for player 0 and player 1, respectively. $\rightarrow \subseteq S \times (\varSigma _0 \cup \varSigma _1) \times S$ is a set of labeled transitions satisfying the following conditions:

Absence of deadlock: for each $s\in S$ there exist $\sigma \in \varSigma _0 \cup \varSigma _1$ and $s'\in S$ s.t. $s \xrightarrow {\sigma } s'$; such an action $\sigma$ is said *enabled* in state *s*;
Alternation: if $s \xrightarrow {\sigma } s'$ for some $\sigma \in \varSigma _0 \cup \varSigma _1$ then $\sigma \in \varSigma _i$ iff $s\in S_i$ and $s'\in S_{1-i}$;
Determinacy of $\varSigma _0$ moves:
if $s\in S_0$ and $s \xrightarrow {\sigma } s_1$ and $s \xrightarrow {\sigma } s_2$ then $s_1=s_2$. $\lhd$

The state space is required to be deadlock-free and bipartite with respect to the transitions, which thus alternate between $S_0$ and $S_1$ states. Furthermore, the actions of player 0 are from $\varSigma _0$ and deterministic, while all actions of player 1 in $\varSigma _1$ can be lumped together into a non-deterministic *u* action, since we are interested in synthesizing a winning strategy merely for player 0, who models the controller. Hence for brevity, we will in the sequel drop $\varSigma _1$ in *G* while denoting $\varSigma _0$ simply as $\varSigma$.

The game is played by a controller (player 0, ego) against an environment (player 1, alter) in turns. Starting from $s = s_0$ and in each second turn, the controller chooses an action $\sigma \in \varSigma$ that is enabled in the current state *s*. By $s \xrightarrow {\sigma } s'$, this leads the game to a unique successor state $s' \in S_1$. From $s'$, it now is the environment’s turn to select an action, which it does by selecting a successor state $s''\in S_0$ with $s'\xrightarrow {u} s''$. As $s''$ again is a position controlled by player 0, the game alternates between moves of player 0 (the controller) and player 1 (the environment) forever, leading to the definition of *infinite play* as in Definition 2.

#### Remark 1

Definition 1 assumes that the game graph is bipartite, as is in [29]. We remark, however, that this assumptions is non-substantial: the game graph defined in Definition 1 can be reduced equivalently to that used in [10] with the bipartition assumption (and the determinacy of $\varSigma$ moves) dropped. For instance, a bipartite graph consisting of transitions $s \xrightarrow {\sigma } s'$, $s' \xrightarrow {u} s''_1$ and $s' \xrightarrow {u} s''_2$ can be reduced to a non-partite one with transitions $s \xrightarrow {\sigma } s''_1$ and $s \xrightarrow {\sigma } s''_2$, where ego chooses an action in $\varSigma$ and then alter resolves non-determinism by choosing the successor state. $\lhd$

#### Definition 2

*(Infinite play)* A *play* on game graph $G = \langle S, s_0, S_0, S_1, \varSigma , \rightarrow \rangle$ is an infinite sequence $\pi = \pi _0 \sigma _0 \pi _1 \ldots \sigma _{n-1} \pi _n \sigma _n \ldots$ satisfying $\pi _0 = s_0$ and $\forall i \in {\mathbb {N}}:\pi _i \xrightarrow {\sigma _i} \pi _{i+1}$. $\lhd$

To define a game over the game graph, which merely determines the possible positions and moves in the game, the game graph comes with an associated *winning condition*. In a *safety game*, this is a set of *unsafe positions* ${{\mathcal {U}}} \subseteq S$ and the controller loses (and thus the environment wins) the play $\pi$ as soon as the play reaches an unsafe state $\pi _i \in {\mathcal {U}}$. Conversely, the controller wins (and the environment loses) iff the play $\pi$ goes on forever without ever visiting ${\mathcal {U}}$.

#### Definition 3

*(Regular two-player safety game)* A *(regular) two-player safety game* is of the form $G = \langle S, s_0, S_0, S_1, \varSigma , {\mathcal {U}}, \rightarrow \rangle$, where $G' = \langle S, s_0, S_0, S_1, \varSigma , \rightarrow \rangle$ is a finite game graph and ${{\mathcal {U}}} \subseteq S$ is a set of *unsafe positions*. $\varPi (G)$ denotes the set of plays over the underlying game graph $G'$. The play $\pi _0 \sigma _0 \pi _1 \ldots \in \varPi (G)$ is *won by player 0* iff $\forall i\in {\mathbb {N}}:\pi _i \not \in {{\mathcal {U}}}$ and *won by player 1* otherwise. $\lhd$

The objective of the controller in a safety game thus is to always select actions avoiding unsafe states, while the hostile or just erratic environment would try to drive the game to a visit of an unsafe state by picking adequate successor states on *u* actions.

For a given play $\pi \in \varPi (G)$, its *prefix* up to position $\pi _n$ is denoted by $\pi (n)$. This prefix thus is the finite sequence $\pi (n)=\pi _0 \sigma _0 \pi _1 \ldots \sigma _{n-1} \pi _n$, whose *length* is $|\pi (n)| = n+1$ and whose *last* element is $\mathtt {Last}(\pi (n)) = \pi _n$. The set of prefixes of all plays in $\varPi (G)$ is denoted by $\mathtt {Pref}(G)$, in which we denote those ending in a controller state by $\mathtt {Pref}_c(G) = \{\rho \in \mathtt {Pref}(G) \mid \mathtt {Last}(\rho ) \in S_0\}$. Likewise, $\mathtt {Pref}_e(G) = \{\rho \in \mathtt {Pref}(G) \mid \mathtt {Last}(\rho ) \in S_1\}$ marks prefixes of plays ending in environmental positions.

For a game $G = \langle S, s_0, S_0, S_1, \varSigma , {\mathcal {U}}, \rightarrow \rangle$, a *strategy for the controller* is a mapping $\xi :\mathtt {Pref}_c(G) \rightarrow \left( 2^{\varSigma }{\setminus }\{\emptyset \}\right)$ such that all $\sigma \in \xi (\rho )$ are enabled in $\mathtt {Last}(\rho )$ and $\xi (\rho )\ne \emptyset$ for any $\rho \in \mathtt {Pref}_c(G)$. The *outcome* of the strategy $\xi$ in *G* is defined as $O(G,\xi ) = \{\pi = \pi _0 \sigma _0 \pi _1 \ldots \in \varPi (G) \mid \forall i \in {\mathbb {N}}:\sigma _{2i} \in \xi (\pi (2i)) \}$ and denotes all plays possible when player 0 respects strategy $\xi$ while player 1 plays arbitrarily.

#### Definition 4

*(Winning strategy for the controller)* A strategy $\xi$ for the controller in a safety game $G = \langle S, s_0, S_0, S_1, \varSigma , {\mathcal {U}}, \rightarrow \rangle$ is *winning for the controller* (or just *winning* for short) iff $\forall \pi = \pi _0 \sigma _0 \pi _1 \ldots \in O(G,\xi ) .\, \forall i \in {\mathbb {N}}:\pi _{i} \not \in {\mathcal {U}}$. $\lhd$

A *strategy for the environment* can be defined similarly as being a mapping ${\tilde{\xi }}:\mathtt {Pref}_e(G) \rightarrow \left( 2^{S_0}{\setminus }\{\emptyset \}\right)$ with equivalent well-definedness conditions as above, namely that ${\tilde{\xi }}(\rho )$ is a non-empty subset of the successor states reachable from $\mathtt {Last}(\rho )$ via an environmental player move whenever $\mathtt {Last}(\rho )\in S_1$. It is *winning for the environment* iff all plays consistent with the strategy satisfy the complement of the above winning condition, i.e., eventually visit ${\mathcal {U}}$.

It is a classical result of game theory that such safety games under perfect observation are determined: one of the two players has a sure winning strategy enforcing a win irrespective of the opponent’s particular choice of actions.

#### Theorem 1

(Determinacy [14]) Safety games are determined, i.e., in each safety game $G = \langle S, s_0, S_0, S_1, \varSigma , {\mathcal {U}}, \rightarrow \rangle$ exactly one of the two players has a winning strategy. $\lhd$

We call a (controller) strategy $\xi :\mathtt {Pref}_c(G) \rightarrow 2^{\varSigma }$ *positional* (or *memoryless*) if for any $\rho$ and $\rho ' \in \mathtt {Pref}_c(G)$, $\mathtt {Last}(\rho ) = \mathtt {Last}(\rho ')$ implies $\xi (\rho ) = \xi (\rho ')$. Being positional implies that at any position in a play, the next decision of a controller applying the strategy only depends on the current position in the game graph and not on the history of the play. As a consequence, such a positional strategy can also be described by a function $\xi ' :S_0 \rightarrow 2^{\varSigma }$ that maps every state of the controller in the game to a set of actions to be performed whenever that state is visited. The reduction to positional strategies is motivated by the fact that in delay-free safety games, whenever there exists a winning strategy for the controller, then there also exists a positional strategy for it.

#### Theorem 2

(Existence of positional strategies [9, 29]) Given a two-player safety game *G*, the set of states from which player 0 (player 1, resp.) can enforce a win is computable, and memoryless strategies are sufficient for the winning party. $\lhd$

The construction of memoryless strategies behind Theorem 2 builds on a backward search for the set *E* of states from which a visit in ${\mathcal {U}}$ can be *enforced* by player 1. More precisely, the computation is based on recursively, starting from $E_0 = {{\mathcal {U}}}$, computing the sets $E_i \subseteq S$ from which player 1 can enforce a visit of ${{\mathcal {U}}}$ in at most $i\in {\mathbb {N}}$ steps:

$$\begin{aligned} E_{i+1} = E_i&\cup \{s \in S_1 \mid \exists \sigma \in \varSigma _1 :s \xrightarrow {\sigma } s'~\text {for some}~s' \in E_i\}\\&\cup \{s \in S_0 \mid \forall \sigma \in \varSigma _0 :s \xrightarrow {\sigma } s'~\text {(if defined)}~~\text {for some}~s' \in E_i\} \end{aligned}$$

As *S* is finite this ascending chain of state sets ($E_i$) eventually reaches a fixed-point *E*. If $s_0 \in E$, then the positional winning strategy for player 1 is obtained by admitting in each state from $S_1 \cap E_{i+1}$ exactly those actions leading to a successor in $S_0 \cap E_i$. Otherwise, a positional winning strategy for player 0 applies to any state in $S_0 {\setminus } E$, specifying a transition to a successor state again outside *E* (which exists by the definition of *E*). An algorithmic fixed-point-based construction of positional strategies will be presented in Sect. 3. For more details, we refer the readers to [29].

### Games under delayed control

It is immediately obvious from the fact that memoryless strategies suffice in the above setting, that being able to fully observe the *current* state and to react on it immediately is an essential feature of the above games. In practice, this is often impossible due to delays between sensing the environmental state, computing the control action, submitting it, and it taking effect. In embedded control applications, such delays are induced by latencies in sensing and actuating devices as well as by the communication networks connecting all the components. The winning strategy for the control player 0, if existent, thus cannot resort to the full state history, but only to a proper prefix thereof due to the remainder of the state history becoming visible too late or even arising only after the control action has been submitted. These delays consequently reduce the strength of the control player 0, who suffers from lacking access to recent state information. It is worth noting, however, that the delays do neither increase the strength of the environmental player 1, as she realistically lacks introspection into the local state of the delay-inducing components (sensors, actuators, communication network infrastructure, etc.) and thus cannot exploit the already submitted, yet latent control information residing therein. This is in stark contrast to the Gale-Stewart-game setting investigated by Holtmann, Kaiser, Thomas, Klein, and Zimmermann [15, 16, 19, 20, 33], where the output player is granted perfect introspection and thus a lookahead into the input player’s delayed actions.

**Fig. 1:**
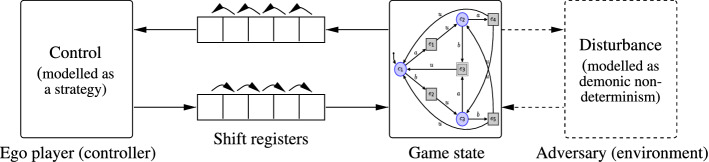
Playing a safety game subject to sequential discrete delay: FIFO-type delays (not necessarily of equal magnitude) arise in the downlink stream through which the controller perceives its environment as well as in the uplink stream through which it actuates effects on the game state

In the simplest possible of a sequence of different settings investigated by us, delayed information is forwarded sequentially and thus in order from the plant to the controller as well as back, equalling two FIFO-buffers or shift-registers (not necessarily of matching lengths) in the two information channels between environment and controller, as depicted in Fig. 1. As can also be inferred from this figure, it does not make any observable difference for the strategy to be played whether delays originate from the downlink connecting the environment to the controller (ego) or the uplink to the controlled plant (adversary) or both; only the total sum of the delays is important: at the interface to the game graph. It is indistinguishable whether the ego player due to a downlink delay of *n* receives at time $t+n$ the state that the game graph has had at time *t*, processes it, delivers its reaction at the next time instant $t+n+1$, which then finally takes effect at time $t+n+1+m$ due to an uplink delay of *m*, or whether any other combination of delays $n'$ and $m'$ with $n'+m'=n+m$ applies. The sequence of events at the interface to the game graph remains unchanged as states observed at time *t* lead to effects at time $t+n+1+m=t+n'+1+m'$; only the absolute time when game states are observed by the controller and reactions are submitted to the network will change, yet the overall turnaround time of $n+1+m=n'+1+m'$ from a game state arising to the corresponding selected action taking effect stays the same, as does consequently the sequence of events observed at the plant’s interfaces. As the sequence of events at the interfaces of the game graph remains unchanged, a strategy consequently is winning under downlink delay *m* and uplink delay *n* iff it is winning under downlink delay $m+n$ and no uplink delay. Likewise, a strategy is winning under downlink delay *m* and uplink delay *n* iff it is winning under no downlink delay and uplink delay $m+n$, provided the uplink channel—here modelled by a shift register—is properly initialized to provide corresponding uplink messages to the environments in steps *n* to $n+m$. We will therefore in the sequel not distinguish between downlink and uplink delays, yet just speak of a single delay of $\delta$ steps. We will freely switch between allocating this delay either within the downlink or the uplink, depending on which choice suits intuition better. Note that in the absence of any observable difference at the interface to the plant, this choice is only a presentational issue without any impact on the theory developed.

For the moment we assume the delay to be constant and equal to $\delta \in {\mathbb {N}}$ steps. The controller would therefore have to decide about the action to be taken after some finite play $\pi _0 \sigma _0 \pi _1 \ldots \pi _{2n}$ already after just seeing its proper prefix $\pi _0 \sigma _0 \pi _1 \ldots \pi _{2n-\delta }$. Furthermore, a constant strategy not dependent on any historic observations would have to be played by the controller initially for the first $\delta$ steps. That motivates the following definition:

#### Definition 5

*(Playing under delay)* Given a delay $\delta \in {\mathbb {N}}$, a *strategy for the controller under delay* $\delta$ is a map $\xi :\mathtt {Pref}_x(G) \rightarrow 2^{\varSigma }$, where $x=c$ if $\delta$ is even and $x=e$ else, together with a non-empty set $\alpha \subseteq \varSigma ^{\lceil \frac{\delta }{2}\rceil }$ of initial action sequences. The *outcome of playing strategy* $(\alpha ,\xi )$ *in* *G* *under delay* $\delta$ is $O(G,\alpha ,\xi ,\delta ) =$

$$\begin{aligned} \left\{ \pi = \pi _0 \sigma _0 \pi _1 \ldots \in \varPi (G) \left| \begin{array}{l@{}} \left( \exists a \in \alpha .\, \forall i \in {\mathbb {N}}:2i<\delta \Rightarrow \sigma _{2 i}=a_i\right) \wedge \\ \left( \forall i \in {\mathbb {N}}:2i \ge \delta \Rightarrow \sigma _{2 i} \in \xi (\pi (2i - \delta ))\right) \end{array} \right. \right\} . \end{aligned}$$

We call the strategy $(\alpha ,\xi )$ *playable* by the controller iff it always assigns permitted moves, i.e., iff for each prefix $\rho = \pi _0 \sigma _0 \pi _1 \ldots \sigma _{2n-1} \pi _{2n}$ of a play in $O(G,\alpha ,\xi ,\delta )$, we have that the set of next actions

$$\begin{aligned} \varSigma _{\rho } = {\left\{ \begin{array}{ll} \{a_n \mid \langle \sigma _0,\sigma _2,\sigma _4,\ldots ,\sigma _{2n-2},a_n \rangle \text { is a prefix of a word in } \alpha \} &{}\quad \text {iff } 2n<\delta ,\\ \xi (\pi (2n - \delta )) &{}\quad \text {iff } 2n\ge \delta \end{array}\right. } \end{aligned}$$

suggested by the strategy is non-empty and contains only actions enabled in $\pi _{2n}$. A strategy $(\alpha ,\xi )$ is *winning (for the controller) under delay* $\delta$ iff it is playable and for each $\pi = \pi _0 \sigma _0 \pi _1 \ldots \in O(G,\alpha ,\xi ,\delta )$, the condition $\forall i \in {\mathbb {N}}:\pi _{i} \not \in {\mathcal {U}}$ holds, i.e., no unsafe state is ever visited when playing the strategy. A strategy $(\alpha ,\xi )$ is *positional* iff it is playable and for any prefixes $\rho$ and $\rho ' \in \mathtt {Pref}_c(G)$ of plays in $O(G,\alpha ,\xi ,\delta )$, $\mathtt {Last}(\rho ) = \mathtt {Last}(\rho ')$ implies that the sets of next actions suggested by the strategy satisfy $\varSigma _{\rho } = \varSigma _{\rho '}$. A winning strategy $(\alpha ,\xi )$ for the controller is *maximally permissive* iff $O(G,\alpha ',\xi ',\delta ) \subseteq O(G,\alpha ,\xi ,\delta )$ for every winning strategy $(\alpha ',\xi ')$ for the controller.

The definition of a *(winning) strategy for the environment* remains unchanged *under delay* $\delta$, as lacking introspection into the state of the components inducing the delay (cf. Fig. 1), the environment still has to base its strategy on just the current (without lookahead) history of actions and game-graph state. A strategy for the environment thus is a mapping ${\tilde{\xi }}:\mathtt {Pref}_e(G) \rightarrow 2^{S_0}$ assigning as ${\tilde{\xi }}(\rho )$ a non-empty subset of the successor states reachable from $\mathtt {Last}(\rho )$ via an environmental player move whenever $\mathtt {Last}(\rho )\in S_1$. It is winning for the environment iff all plays consistent with the strategy ${\tilde{\xi }}$ eventually visit ${\mathcal {U}}$. $\lhd$

Playing under a delay of $\delta$ thus means that for a play $\pi = \pi _0 \sigma _0 \pi _1 \ldots$, the choice of actions suggested by the winning strategy at state $\pi _{2 i}$ has to be pre-decided at state $\pi _{2i - \delta }$ for any $i \ge \lceil \frac{\delta }{2}\rceil$ and decided without recourse to positional information for the first $\delta -1$ steps. Playing under delay 0 is identical to playing a safety game under complete positional information.

From Definition 5 it is obvious that existence of a (delay-free) winning strategy in the complete information game *G* is a necessary, yet not sufficient condition for existence of a strategy that is winning under a delay of $\delta > 0$. Likewise, existence of a strategy winning under some relatively small delay $\delta$ is a necessary, yet not sufficient condition for existence of a strategy that is winning under a delay of $\delta ' > \delta$: having a strategy that wins for $\delta '>\delta$, the strategy that wins for $\delta '$ can successfully be played for $\delta$ also by simply waiting $\delta '-\delta$ steps before implementing the respective control action.

Intuitively, a maximally permissive winning strategy in Definition 5 subsumes the behavior of every winning strategy (cf. [4]). The maximal permissiveness consequently admits non-deterministic strategies, i.e., the controller non-deterministically selects from the set of all guaranteed to be winning actions upon decisions, instead of committing itself to exactly one particular action. Such non-deterministic strategies come with the advantage of avoiding the implementation bias of selecting a particular action, which would likely impede compositionality of strategies. As we need to combine different strategy parts in our incremental synthesis algorithm, we need such compositionality. Insisting on maximal permissiveness of strategies therefore guarantees the completeness of our incremental synthesis algorithm to be proposed, as shown later in the proof of Theorem 3, since the algorithm can always find a winning strategy under delay for the controller if there exists one, by incrementally hardening the maximally permissive strategy against larger and larger delays.

#### Lemma 1

Let $G = \langle S, s_0, S_0, S_1, \varSigma , {\mathcal {U}}, \rightarrow \rangle$ be a safety game. If the controller has no winning strategy under delay $\delta \in \mathbb {N}$, then it has no winning strategy under any delay $\delta ' \in \mathbb {N}$ with $\delta ' \ge \delta$. $\lhd$

#### Proof

We prove the contraposition that if *G* has a winning strategy $(\alpha ',\xi ')$ under delay $\delta '$, then *G* has a winning strategy $(\alpha ,\xi )$ under delay $\delta \le \delta '$ too. This however is intuitively obvious: we can apply the winning strategy that computes an appropriate reaction for delay $\delta '$ and delay its action by further $\delta '-\delta$ steps as follows. If $(\alpha ',\xi ')$ is the winning strategy corresponding to delay $\delta '$ then $(\alpha ,\xi )$ with $\alpha = \{a'(\lceil \delta /2\rceil ) \mid a' \in \alpha '\}$ and

$$\begin{aligned} \xi (\pi (2n)) = {\left\{ \begin{array}{ll} a'_n &{}\quad \text {iff } 2n < \delta ',\\ \xi '\left( \pi \left( 2n -(\delta '-\delta )\right) \right) &{}\quad \text {iff } 2n \ge \delta '\\ \end{array}\right. } \end{aligned}$$

wins *G* under delay $\delta$. $\square$

Before we move on to complex models of delay including out-of-order delivery of actions, we first explore the above scenario of playing under strictly sequential delay with guaranteed in-order delivery of the delayed information as captured by Definition 5. We will show in Sect. 4 how the setting can be extended to cater for out-of-order delivery and bounded message loss, which are present in many practical applications of networked control.

### Insufficiency of memoryless strategies

Recall that in safety games with complete information, the existence of a winning strategy for the controller, i.e., for player 0, implies existence of a memoryless strategy for player 0. For games with delayed information, however, memoryless strategies are not powerful enough, as the following example demonstrates:

#### Example 1

**Fig. 2:**
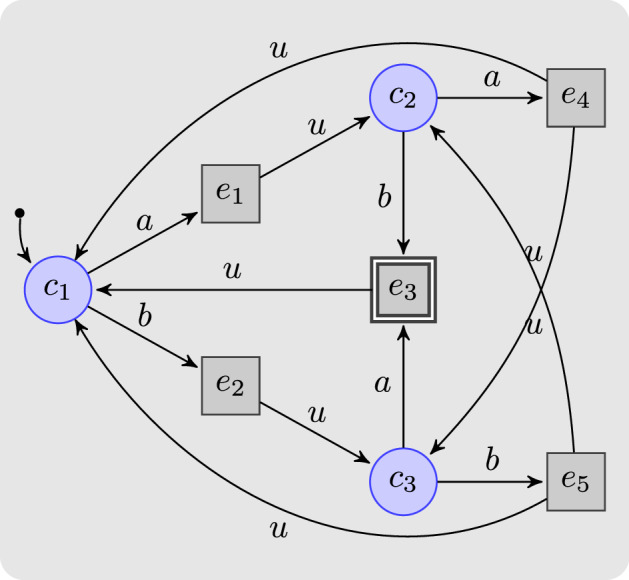
A safety game winnable with memoryless strategies for delay $\delta \le 1$, yet not beyond

Consider the safety game $G = \langle S, s_0, S_0, S_1, \varSigma , {\mathcal {U}}, \rightarrow \rangle$ shown in Fig. 2, where $S = S_0 \cup S_1$, $S_0 = \{c_1, c_2, c_3\}$, $S_1 = \{e_1, e_2, e_3, e_4, e_5\}$, $s_0 = c_1$, $\varSigma = \{a, b\}$, and ${\mathcal {U}}= \{e_3\}$. Player 0 can obviously win this safety game if no delay is involved.

Now consider a memoryless strategy with $\xi ' :S_0 \rightarrow 2^{\varSigma }$ for the controller under delay 2. We obviously need $\xi '(c_2) = \{b\}$, indicating that the controller executes *b* two steps later at either $c_1$ or $c_3$, as action *a* at $c_3$ would yield the unsafe state $e_3$. Analogously, $\xi '(c_3) = \{a\}$ is necessary since *a* always leads to a safe state two steps later at either $c_1$ or $c_2$. At $c_1$, however, the controller has to draw a pre-decision for both $c_2$ and $c_3$. If the controller picks *a* (or *b*) at $c_1$ without knowing whether it will move towards $c_2$ or $c_3$, then two steps later at $c_3$ ($c_2$, resp.) it executes the unsafe action *a* (*b*, resp.). For a win, extra memory keeping track of the historic sequence of actions is necessary such that the controller can retrieve the still latent action previously emitted at $c_2$ or $c_3$ and can thus determine whether it will next visit $c_2$ or $c_3$ from $c_1$. $\lhd$

The above example shows that memoryless strategies are generally insufficient for winning a safety game under delays. A straightforward generalization of the situation shown in Fig. 2, namely deeply nesting triangles of the shape spanned by $c_1$, $c_2$, and $c_3$, demonstrates that the amount of memory needed will in worst case be exponential in the delay. Any reduction of delayed safety games to safety games under complete information—i.e., to delay-free safety games—will have to introduce a corresponding blow-up of the game graph. We will next elaborate such a reduction.

### Reduction to delay-free games

**Fig. 3:**
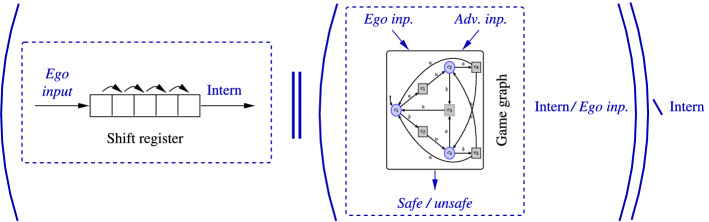
An illustration of the reduction to delay-free games. Direct input of the ego player to the game graph is replaced by an input to a shift register, whose output is connected to the game graph via an internal channel. Note that the shift register represents an automaton of size exponential in the magnitude of the delay such that game graph resulting from the synchronous product with the original game graph incurs an exponential blow-up. Here, “Intern” abbreviates internal actions

As playing a game under delay $\delta$ amounts to pre-deciding actions $\delta$ steps in advance, the problem of finding a winning strategy for the controller in *G* that wins under delay $\delta$ can be reduced to the problem of finding an undelayed winning strategy for the controller in a related safety game which is obtained by building a synchronous product of the original game graph with a shift register implementing the delay by queueing the latent actions in FIFO manner. Within this derived game depicted in Fig. 3, actions submitted by the controller have to queue in a FIFO buffer of length $\delta$ before they affect the game state. The controller in its first step can thus preload that FIFO buffer (which requires an alphabet extension for the controller such that it can select an initial FIFO contents in a single sweep) and will then always submit its subsequently single action $\sigma \in \varSigma$ to the tail of the buffer while the environment retrieves player-0 actions from the head of the buffer such that actions get delayed by the FIFO-buffer length. This reduction can be formalized as follows:

#### Lemma 2

Let $G=\langle S, s_0, S_0, S_1, \varSigma , {\mathcal {U}}, \rightarrow \rangle$ be a safety game and $\delta \in {\mathbb {N}}$ a delay. Then the controller has a strategy that wins *G* under delay $\delta$ iff the controller has a winning strategy in the game ${\widehat{G}}=\langle S', s_0', S_0', S_1', \varSigma \cup \varSigma ^{\lceil \frac{\delta }{2}\rceil }, {\mathcal {U}}', \rightarrow '\rangle$ given by

1. $S' = \left( S \times \varSigma ^{\lceil \frac{\delta }{2}\rceil }\right) \uplus \{s_0'\} \uplus \left( \{s_0'\} \times \varSigma ^{\lceil \frac{\delta }{2}\rceil }\right)$, where $\uplus$ denotes disjoint union, $S_0' = \left( S_0 \times \varSigma ^{\lceil \frac{\delta }{2}\rceil }\right) \cup \{s_0'\}$, and $S_1' = \left( S_1 \times \varSigma ^{\lceil \frac{\delta }{2}\rceil }\right) \cup \left( \{s_0'\} \times \varSigma ^{\lceil \frac{\delta }{2}\rceil }\right)$,
2. $$s \overset{\sigma }{\rightarrow '} s'$$ iff
  $$\begin{aligned} \begin{aligned}&\, s=s_0' \wedge \sigma =a_1\ldots a_n \in \varSigma ^{n} \wedge s'= (s_0',a_1\ldots a_n)&\mathrm{[trans.~from~initial~state~}s_0'{~\mathrm{to}~}(s_0',\alpha )]\\ \vee&\, s=(s_0',\alpha ) \wedge \sigma =u\wedge s'=(s_0,\alpha )&\mathrm{[trans.~ from~}(s_0',\alpha )~\mathrm{to }~(s_0,\alpha )]\\ \vee&\, s=({\hat{s}},a_1\ldots a_n) \wedge {\hat{s}}\in S_0 \wedge \sigma \in \varSigma \wedge {\hat{s}} \xrightarrow {a_1} {\hat{s}}' \wedge s'= ({\hat{s}}',a_2\ldots a_n \sigma )&\mathrm{[trans.~ from~}S_0'~\mathrm{to }~S_1']\\ \vee&\, s=({\hat{s}},\alpha ) \wedge {\hat{s}}\in S_1 \wedge \sigma = u \wedge {\hat{s}} \xrightarrow {u} {\hat{s}}' \wedge s'= ({\hat{s}}',\alpha )&\mathrm{[trans.~ from~}S_1'~\mathrm{to }~S_0'] \end{aligned} \end{aligned}$$
  where $n=\frac{\delta }{2}$ if $\delta$ is even and $n=\frac{\delta +1}{2}$ if $\delta$ is odd, i.e., $n = \lceil \frac{\delta }{2}\rceil$.
3. $${\mathcal {U}}' = {\mathcal {U}} \times \varSigma ^{\lceil \frac{\delta }{2}\rceil }$$. $$\lhd $$

#### Proof

We first concentrate on the case of even delay $\delta$. For an even delay $\delta$, game ${\widehat{G}}$ simulates playing *G* under delay $\delta$ by first forcing the controller to guess an initial action sequence $\alpha \in \varSigma ^{\frac{\delta }{2}}$ and then maintains a shift register of player-0 actions. It subsequently determines actions of player 0 by the head of the shift register while appending fresh actions to its tail, thus delaying the effect of player-0 actions by $\delta$ steps. As each action that thus comes to effect has been decided $\delta$ time units ago, this is equivalent to deciding actions at step *i* based on the play prefix $\pi (i-\delta )$, as a strategy under delay would have to. Consequently, a winning strategy for the controller in this safety game ${\widehat{G}}$ exists iff a strategy winning for the controller in *G* under delay $\delta$ exists.

For the case of odd delay $\delta =2k+1$, we observe that the move from a state at $\pi _{i-\delta -1}$ to $\pi _{i-\delta }$ is a player-0 action if *i* itself is an even position, i.e., under control of the controller. If playing a deterministic strategy, which obviously is as powerful as playing potentially non-deterministic strategies, the controller consequently cannot gain any additional information from being able to observe the play prefix $\pi (i-\delta )$ rather than just the shorter prefix $\pi (i-\delta -1)$. The problem of finding a strategy under odd delay $\delta$ thus is equivalent to that of finding a strategy for even delay $\delta +1$, which warrants using reduction to the same safety game ${\widehat{G}}$ in both cases. $\square$

The essential idea of the above reduction is to extend the game graph by a synchronous product with a shift register appropriately delaying the implementation of the control action decided by the controller. The blow-up in graph size incurred is by a factor $|\varSigma |^{\lceil \frac{\delta }{2}\rceil }$ and thus exponential in the delay. It is obvious that due to this, a winning strategy for the controller in the delayed game can, if existent, be synthesized with $|\varSigma |^{\lceil \frac{\delta }{2}\rceil }$ memory.

Note that the above reduction to delay-free safety games does not imply that games under delay are determined, as the reduction to delay-free games from Lemma 2 does not apply symmetrically for the environment. Rather, due to it lacking introspection into the states of delay-inducing components, the environment interacts with the game graph in an unaltered fashion. This implies that the environment’s strategies as well as its winning strategies remain unchanged (cf. the definition of (winning) strategy for the environment in Definition 5) under delay. For the environmental player, the reduction of a safety game under delay to a corresponding delay-free game thus is trivial: it just recovers the underlying delay-free game. As consequently, delays impact the strength of the control player while leaving the strength of the environmental player unchanged, situations where none of the two players owns a winning strategy may arise. Delayed games may thus be undetermined, as demonstrated by the following simple example: Consider a simple guessing game, where player 1 suggests in each of her moves either a “head” or a “tail” and player 0 in the next step has to repeat that exact guess by revealing a corresponding “head” or “tail” action, losing as soon as he fails to properly repeat the previous guess. In this guessing game, player 0 has a sure winning strategy under delay 0, as he just has to repeat the most recent observation. Yet none of the two players has one under any strictly positive delay: while player 1 could enforce a win with probability 1 in a probabilistic setting by just playing a random sequence, she cannot enforce a win in the qualitative setting where player 0 may just be lucky to draw the right guesses throughout. Determinacy is only obtained if one of the players is granted a lookahead equivalent to the other’s delay, as in the settings of Hosch and Landweber [17] and the Gale-Stewart setting of Holtmann, Kaiser, Thomas, Klein, and Zimmermann [15, 16, 20]. Such lookahead does not, however, correspond to any physical reality in distributed control, where both players are subject to the same positive end-to-end latency (i.e., delay) in their mutual feedback loop. The FIFO queue thus is opaque to and thus hinders both players, rather than granting advantage to player 0 by providing introspection into actions scheduled by player 1 for the future, as in [20].

## Synthesizing controllers

As stated above, controller synthesis for games under delay $\delta$ can be obtained using a reduction to a delay-free safety game involving the introduction of a shift register. The exponential blow-up incurred by this reduction, however, seems impractical for any non-trivial delay. We therefore present a novel incremental synthesis algorithm, which starts from synthesizing a winning strategy for the underlying delay-free safety game (with delay $k = 0$) and then incrementally hardens the strategy against larger and larger delays (till the final delay of interest $k=\delta$, if existent), thus avoiding explicit reductions. We further optimize the algorithm by pruning the otherwise exponentially sized game graph after each such hardening step: as controllability (i.e., the controller wins) under delay *k* is a necessary condition for controllability under delay $k'>k$, each state already uncontrollable under delay *k* can be removed before proceeding to the next larger delay. The algorithm thus alternates between steps extending memory and thus effectively enlarging the game graph underlying the reduction to delay-free safety games, and steps compressing the game graph.

Reflecting the fact that the additional memory is a FIFO memorizing $\lceil \delta /2 \rceil$ latent actions, we will for the sake of clarity of presentation in this section denote our strategies slightly differently from that in Sect. 2: rather than saying a strategy under delay $\delta$ is a pair $(\alpha ,\xi )$ with $\alpha \in \varSigma ^{\lceil \delta /2\rceil }$ and $\xi :\mathtt {Pref}_x(G) \rightarrow 2^{\varSigma }$, we denote strategies as pairs $(\alpha ,{\hat{\xi }})$ with $\alpha \in \varSigma ^{\lceil \delta /2\rceil }$ and ${\hat{\xi }}:S_x \times \varSigma ^{\lceil \delta /2\rceil } \rightarrow 2^{\varSigma }$. Such a pair $(\alpha ,{\hat{\xi }})$ corresponds to a delay strategy $(\alpha ,\xi )$ by the recursive equation

$$\begin{aligned} \xi (\pi (n)) = {\hat{\xi }}\left( \pi _n, \langle \xi '(\pi (n-2\delta )), \xi '(\pi (n-2\delta +2))\ldots , \xi '(\pi (n-2)) \rangle \right) \end{aligned}$$

where

$$\begin{aligned} \xi '( \sigma _0 \pi _1 \ldots \sigma _{k-1} \pi _k) = {\left\{ \begin{array}{ll} \xi (\pi _0 \sigma _0 \pi _1 \ldots \sigma _{n-1} \pi _k) &{}\quad \text {iff } k \ge \delta ,\\ \alpha _k &{}\quad \text {else.} \end{array}\right. } \end{aligned}$$

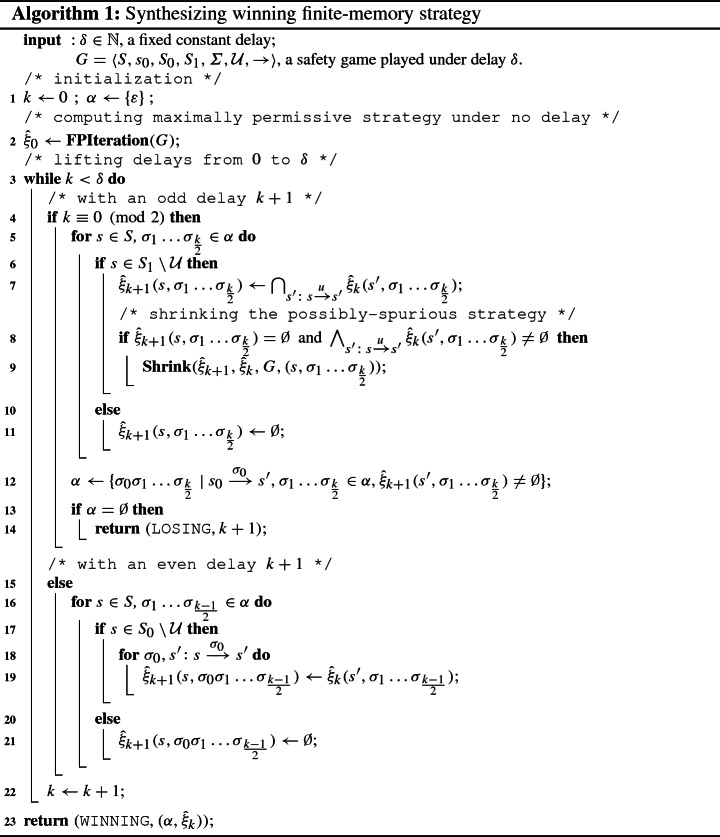


The key idea of the synthesis procedure (Algorithm 1) is to compute a series of finite-memory winning strategies ${\hat{\xi }}_k$ while incrementing delays from $k = 0$ to the final delay of interest $k=\delta$. The algorithm takes as input a delayed safety game $G_\delta$ (notational abbreviation of a safety game *G* played under the fixed delay $\delta$), and returns either ${\texttt {WINNING}}$ paired with a winning strategy $(\alpha ,{\hat{\xi }}_\delta )$ for the controller if $G_\delta$ is controllable, or ${\texttt {LOSING}}$ otherwise with an integer *m* indicating that the winning strategy vanishes at delay $m \le \delta$. Line 2 invokes the classical fixed-point iteration to generate the maximally permissive strategy for the controller in *G* without delay. The procedure **FPIteration** (Algorithm 2) first conducts a backward fixed-point iteration computing the set *E* of states from which a visit to ${\mathcal {U}}$ can be enforced by the alter player 1 [29]. The maximally permissive strategy for the controller is then obtained by admitting in each state from $S_0{\setminus } E$ exactly those actions leading to a successor in $S_1{\setminus } E$. Then the delays are lifted from $k = 0$ to $\delta$ by a $\mathtt {while}$ loop in line 3, and within each iteration of the loop the strategy ${\hat{\xi }}_{k+1}$ is computed based on its predecessor ${\hat{\xi }}_{k}$ as follows:

1. If $k+1$ is an odd delay, the controller needs to make pre-decisions at safe states of the environment, namely at each $s \in S_1 {\setminus } {\mathcal {U}}$. The controller needs to pre-decide at *s* a set of actions that are safe to perform at any of the possible successors $s' \in \mathtt {Succ}(s)$, irrespective of which successor state $s'$ actually happens to arise. For each such successor state, a corresponding set of winning actions has however already been computed in the strategy ${\hat{\xi }}_k(s',\cdot )$. In line 7, we therefore compute ${\hat{\xi }}_{k+1}(s,\rho )$ as the intersection of the ${\hat{\xi }}_{k}(s',\rho )$ for all $s' \in \mathtt {Succ}(s)$ with the same history sequence of actions $\rho$. The derived strategy can be spurious however, inasmuch as the intersection may itself be empty or lead to successor states with an empty strategy, i.e., which are uncontrollable. At line 9 we therefore remove all uncontrollable predecessors of freshly unwinnable states by a **Shrink** procedure depicted in Algorithm 3, which will be explained below.
2. In case of an even delay $k+1$, the controller needs to make pre-decisions at safe states of its own, i.e. at each $s \in S_0 {\setminus } {\mathcal {U}}$. In contrast to an intersection in the odd case, the controller can inherit the winning strategy ${\hat{\xi }}_k(s',\rho )$ directly from each successor $s'$ of *s*. However, we have to prepend, if $s \xrightarrow {\sigma _0} s'$, the action $\sigma _0$ to the history sequence $\rho$ to record the choice in the shift register (line 19).

The synthesis aborts at line 14 if the controller does not have any actions available at the initial state $s_0$, declaring ${\texttt {LOSING}}$ at $k+1$ where the winning strategy vanishes. Otherwise, the algorithm continues and eventually produces a winning strategy ${\hat{\xi }}_\delta$ for the controller in *G*.
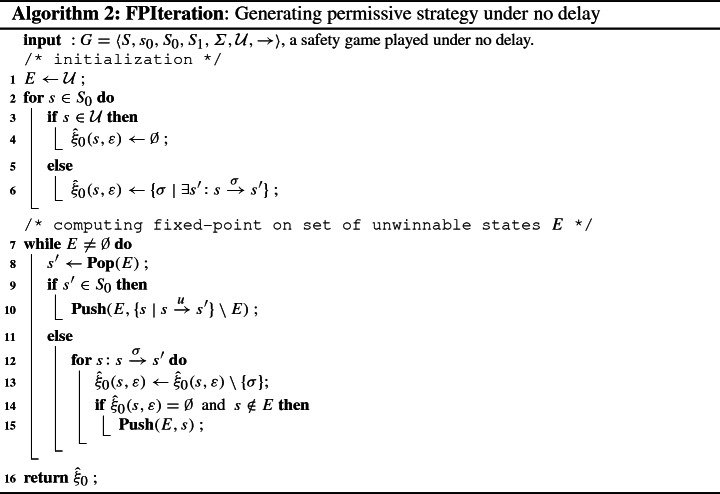

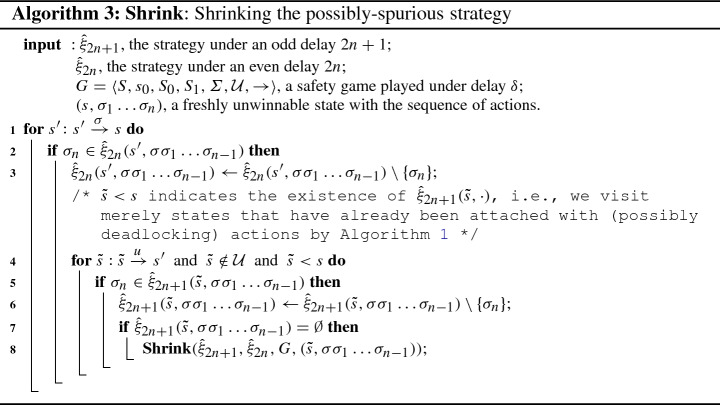


Only when a fresh unwinnable state *s* for the controller is detected (line 8), the **Shrink** function (Algorithm 3) will be launched to carry out two tasks in a recursive manner: (1) it traverses the graph backward and removes from the current strategy all the actions that may lead the play to this unwinnable state, and consequently (2) it gives a state-space pruning that removes all states no longer controllable under the given delay before proceeding to the next larger delay. The latter accelerates synthesis, while the former is a key ingredient to the correctness of Algorithm 1, as can be seen from the proof of Theorem 3: it avoids “blind alleys” where locally controllable actions run towards subsequent unwinnable states.

The worst-case complexity of Algorithm 1 follows straightforwardly as $O(\delta \cdot |S_0| \cdot |S_1| \cdot |\varSigma |^{\lfloor \frac{\delta }{2}\rfloor })$, as is the case for the reduction to a delay-free safety games. In practice, the advantage however is that we avoid explicit construction of the graph of the corresponding delay-free game, which yields an exponential blow-up, and interleave the expansion by yet another shift-register stage with state-set shrinking removing uncontrollable states.

### Theorem 3

(Correctness and Completeness) Algorithm 1 always terminates. If its output is $(\texttt {WINNING },(\alpha ,{\hat{\xi }}))$ then $(\alpha , {\hat{\xi }})$ is a maximally permissive winning strategy of $G_\delta$; otherwise, when $(\texttt {LOSING }, k+1)$ is the output of the algorithm, $G_\delta$ has no winning strategy. $\lhd$

### Proof

Termination is trivially guaranteed by the strictly increasing index *k* bounded by the final delay of interest $\delta$. For convenience, we define the union of two maps ${\hat{\xi }}_1, {\hat{\xi }}_2 :S \times \varSigma ^{\lfloor \frac{\delta }{2}\rfloor } \rightarrow 2^{\varSigma }$ as ${\hat{\xi }}_1\cup {\hat{\xi }}_2 :S \times \varSigma ^{\lfloor \frac{\delta }{2}\rfloor } \rightarrow 2^{\varSigma }$ by $({\hat{\xi }}_1\cup {\hat{\xi }}_2)(s,\alpha )={\hat{\xi }}_1(s,\alpha )\cup {\hat{\xi }}_2(s,\alpha )$ $\forall s\in S$, $\alpha \in \varSigma ^{\lfloor \frac{\delta }{2}\rfloor }$. It then follows that if $(\alpha ,{\hat{\xi }}_1)$ and $(\alpha ,{\hat{\xi }}_2)$ are both winning strategies of a game with delay $\delta$, then $(\alpha ,{\hat{\xi }}_1\cup {\hat{\xi }}_2)$ is also a winning strategy. This fact allows us to specify for any $\alpha \in \varSigma ^{\lfloor \frac{\delta }{2}\rfloor }$ the maximally permissive winning strategy w.r.t. $\alpha$ as $(\alpha , \cup {\hat{\xi }})$, where the union is over all such ${\hat{\xi }}$’s that $(\alpha ,{\hat{\xi }})$ is a winning strategy with delay $\delta$.

Now we prove that with output $({\texttt {WINNING}},\alpha , {\hat{\xi }}_\delta )$, the strategy $(\alpha ,{\hat{\xi }}_\delta )$ is actually a maximally permissive winning strategy of game $G_\delta$. We prove by induction on *k* that during execution of the algorithm, $(\alpha ,{\hat{\xi }}_k)$ is always a maximally permissive winning strategy of the game with delay *k*. The initial case of $k=0$ is guaranteed by $\mathbf{FPIteration }$ in line 2 of Algorithm 1 (cf. Fact 3.1 in [4] and the proof thereof), and the induction from *k* to $k+1$ is achieved by two steps. First, we prove that $(\alpha ,{\hat{\xi }}_{k+1})$ is a winning strategy. It suffices to prove the fact $\emptyset \ne O(G,\alpha ,{\hat{\xi }}_{k+1},k+1)\subseteq O(G,\alpha ,{\hat{\xi }}_k,k)$, which is demonstrated in the following two cases:

1. For an even *k*, the strategy $(\alpha , {\hat{\xi }}_{k+1})$ is playable, as for any path $\pi =\pi _0\sigma _0\pi _1\sigma _1\ldots$ obtained under $(\alpha , {\hat{\xi }}_{k+1})$, ${\hat{\xi }}_{k+1}(\pi _{2i+1},\sigma _{2i+2}\sigma _{2i+4}\ldots \sigma _{2i+k})\ne \emptyset$; otherwise, the configuration $(\pi _{2i+1},\sigma _{2i+2}\ldots \sigma _{2i+k})$ will be removed by the **Shrink** procedure in line 9 and thus cannot be reached under the strategy. Furthermore, the assignment in line 7 implies that for any play $\pi _0\sigma _0\pi _1\sigma _1\ldots \in \varPi (G)$,
  $$\begin{aligned} \alpha _{2i}\in {\hat{\xi }}_{k+1}(\pi _{2i-k-1},\sigma _{2i-k}\sigma _{2i-k+2}\ldots \sigma _{2i-2}) \Rightarrow \alpha _{2i}\in {\hat{\xi }}_k(\pi _{2i-k},\sigma _{2i-k} \ldots \sigma _{2i-2}), \end{aligned}$$
  thus we have $O(G,\alpha ,{\hat{\xi }}_{k+1},k+1)\subseteq O(G,\alpha ,{\hat{\xi }}_k,k)$. So, all the outcomes are safe from the induction.
2. For an odd *k*, playing under $(\alpha ,{\hat{\xi }}_{k+1})$ is as the same as playing under $(\alpha ,{\hat{\xi }}_k)$, since $O(G,\alpha ,\xi _{k+1},k+1)=O(G,\alpha ,\xi _k,k)$ can be verified from the assignment in line 19. So, $(\alpha ,{\hat{\xi }}_{k+1})$ is guaranteed to be a winning strategy, as it is the same as $(\alpha ,{\hat{\xi }}_k)$.

Second, the maximality of $(\alpha ,{\hat{\xi }}_{k+1})$ can be argued by the maximality of $(\alpha ,{\hat{\xi }}_k)$, together with the following fact: for any winning strategy $(\alpha ,{\hat{\xi }}_{k+1}')$ for an odd delay $k+1$, a winning strategy $(\alpha ',{\hat{\xi }}_k')$ for delay *k* can be constructed by

1 $$\begin{aligned} \alpha '=\{\alpha _0\ldots \alpha _{\frac{k}{2}-1}\mid \alpha _0\ldots \alpha _{\frac{k}{2}}\in \alpha \}\, \text {and} \, \, {\hat{\xi }}_k'(s',\sigma _1\ldots \sigma _\frac{k}{2})={\hat{\xi }}_{k+1}'(s,\sigma _1\ldots \sigma _{\frac{k}{2}}), \end{aligned}$$

where $s\xrightarrow {u}s'$, and the maximality of the winning strategy is preserved.

To prove that with output $({\texttt {LOSING}}, k)$ the game has no winning strategy even for delay *k*, we note that *k* should be an odd number in this case. Suppose that there is a winning strategy $(\alpha ,{\hat{\xi }}_k^\prime )$ for delay *k*. We can then construct a winning strategy $(\alpha ',{\hat{\xi }}_{k-1}^\prime )$ for delay $k-1$ as by Eq. (1). It is easy to check that ${\hat{\xi }}_{k-1}\cup {\hat{\xi }}_{k-1}^\prime \ne {\hat{\xi }}_{k-1}$, which contradicts to the maximality of ${\hat{\xi }}_{k-1}$. $\square$

### Example 2

Consider the safety game *G* under delayed information in Fig. 2. The series of finite-memory winning strategies produced by Algorithm 1 is:

$$\begin{aligned} {\hat{\xi }}_0 (c_1,\varepsilon )&= \{a, b\},&{\hat{\xi }}_0 (c_2,\varepsilon )&= \{a\},&{\hat{\xi }}_0 (c_3,\varepsilon )&= \{b\}.\\ {\hat{\xi }}_1 (e_1,\varepsilon ) = \{a\},&\quad {\hat{\xi }}_1 (e_2,\varepsilon ) = \{b\},&{\hat{\xi }}_1 (e_3,\varepsilon )&= \emptyset ,&\!\!\!\!\!\!{\hat{\xi }}_1 (e_4,\varepsilon ) = \{b\},&\quad {\hat{\xi }}_1 (e_5,\varepsilon ) = \{a\}.\\ {\hat{\xi }}_2 (c_1,a)&= \{a\},&{\hat{\xi }}_2 (c_2,a)&= \{b\},&{\hat{\xi }}_2 (c_3,a)&= \emptyset ,\\ {\hat{\xi }}_2 (c_1,b)&= \{b\},&{\hat{\xi }}_2 (c_2,b)&= \emptyset ,&{\hat{\xi }}_2 (c_3,b)&= \{a\}. \end{aligned}$$

Winning strategies for the controller vanish when the delay reaches 3. $\lhd$

### Upper bounding delay where winning strategy vanishes

From the structure of Algorithm 1 it is obvious that there must exist a magnitude of delay beyond which the strategy does not change any more, as the algorithm essentially just builds intersections of finitely many different action sets present in the maximally permissive memoryless strategy for the original safety game. As there are only finitely many combinations of these sets, the strategy has to stabilize eventually, implying that it either vanishes at some delay (which is detected by Algorithm 1) or there is a strategy suitable for arbitrarily large delay, which furthermore is eventually constructed by the synthesis algorithm. Klein and Zimmermann have shown in [19, Theorem 2] that there exist safety game graphs that may be lost by the input player (corresponding to the controller in our setting) only under a delay that is, up to a constant factor, exponential in the size of the game graph. This bound, however, constitutes a lower bound on the worst-case and may not be indicative for game graphs featuring a different structure than these hard instances. We therefore complement this result here by a variant that refers to the particular structure of the game graph under investigation.

From the way Algorithm 1 combines the original action sets from the memoryless strategy along the transitions in the game graph, it is obvious that in worst case it can take the least common multiple (LCM) of the lengths of all cycles in the game graph plus the maximum lengths of the acyclic paths leading from the initial state into cycles and the acyclic paths leading out of cycles to states in ${{\mathcal {U}}}$ to compute all combinations. As the algorithm is complete in the sense that it computes a delay-resilient strategy for a delay $\delta$ whenever such exists, we therefore conjecture:

#### Conjecture 1

Let $G = \langle S, s_0, S_0, S_1, \varSigma , {\mathcal {U}}, \rightarrow \rangle$ be a safety game and let $\delta$ be the LCM of the lengths of all cycles in the game graph plus the maximum lengths of the acyclic paths leading from the initial state into cycles and the acyclic paths leading out of cycles to states in ${{\mathcal {U}}}$. If *G* has a winning strategy under delay $\delta$ then it also has a winning strategy under any delay $\delta '>\delta$. $\lhd$

## Extension to out-of-order delivery of messages and bounded message loss

From a practical standpoint of networked control, the assumptions may not be justified that all messages between the environment and the controller are actually delivered and that they arrive with a constant delay and thus especially in the order of sending. These assumptions do however underlie our above model of games under delay. The control strategies that are synthesized by Algorithm 1 are consequently only known to be correct under these rather strong assumptions. Within this section we will show that they are equally adequate in settings relaxing these assumptions. The relaxed settings we are looking into are, first, out-of-order delivery with a guaranteed maximal message delay and, second, potential message loss with a fixed bound on the maximum number of consecutive message losses.

Throughout this section, we adopt the assumption that, during the play, any state observed is associated with a *timestamp* $\tau \in {\mathbb {N}}$ indicating when it arose. This convention is necessary as out-of-order delivery or message loss would else prohibit reconstruction of the time instant a reported state pertains to. With the timestamp, the controller is capable of recognizing the order of the action delivery. In particular we denote the current time by $now \in {\mathbb {N}}$.

### Out-of-order delivery

**Fig. 4:**
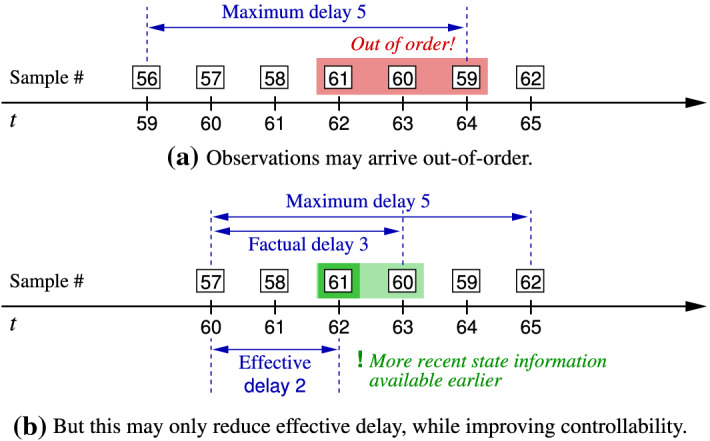
An example of out-of-order delivery of messages. The maximum message delay here is 5, while the factual delay for the state information pertaining to actual time 60 is 3, as that data item arrives at time 63. But as even more recent state information pertaining to time instant $61 > 60$ becomes available already at time instant 62, the effective delay is just 2

In the definition of playing a safety game under delay (Definition 5), we assumed strictly sequential and constant delay, i.e., reliable in-order delivery of the delayed information exactly $\delta$ time units late. While this cannot be guaranteed in many practical applications of networked control, the assumption does not impede applicability of the theory developed so far, as random out-of-order delivery with a maximum delay of $\delta$ has in-order delivery with an exact delay of $\delta$ as its worst-case instance: whenever a data item is delivered out-of-order then it is delivered before $\delta$, implying earlier availability of more recent state information and thus enhanced controllability. In a qualitative setting, solving the control problem for out-of-order delivery with a maximum delay of $\delta$ consequently is—up to the necessity of appropriately delaying reactions to data items arriving early—identical to solving the control problem under in-order delivery with an exact delay of $\delta$, as the latter is the former’s worst case. This fact is illustrated in Fig. 4 and further formalized in Proposition 1.

#### Proposition 1

For a two-player safety game, there exists a winning strategy under out-of-order delivery of state information with a maximum delay of $\delta$ iff there exists a winning strategy under in-order delivery with an exact delay of $\delta$. $\lhd$

#### Proof

The “only if” part is for free, since playing under out-of-order delivery with a maximum delay of $\delta$ subsumes the case of playing under in-order delivery with an exact delay of $\delta$. For the “if” part, it suffices to prove that a winning strategy $(\alpha ,{\hat{\xi }}_\delta )$ for the controller under in-order delivery with an exact delay of $\delta$, if existent, is also a winning strategy for the controller under out-of-order delivery with a maximum delay of $\delta$. For the latter, whenever an observation of the game state $\pi _t$ arrives early at time $\tau <t+\delta$, it can be cached till time $\tau '=t+\delta$, thus facilitating to play the strategy $(\alpha ,{\hat{\xi }}_\delta )$ that expects all data items to arrive with an exact constant delay of $\delta$. Note that due to absence of message loss and the maximum delay being $\delta$, all data items are guaranteed to arrive before or at $\delta$ time units after their generation. The caching of early arrivals (on top of Algorithm 1, with adequate timestamping) thus supplies the strategy $(\alpha ,{\hat{\xi }}_\delta )$ with an uninterrupted sequence of historic states up to $\delta$ time units before $now$. The winning strategy $(\alpha ,{\hat{\xi }}_\delta )$ can thus be applied without any modification. $\square$

Strictly speaking, the discussion in the above proof only dealt with the case of (fixedly bounded, yet not necessarily constant) delays in the downstream link from the environment to the controller, yet not back. But timestamping control actions with the time they ought to be applied and buffering them at the control interfaces of the environment until their expected time of application works the same way. We can consequently conclude that Algorithm 1 solves the problem of computing winning strategies for the out-of-order setting too.

#### Corollary 1

Let *G* be a safety game and let the controller be connected to the environment via channels ensuring a maximum message delay of $\delta$, yet allowing for out-of-order delivery. If all messages are timestamped then Algorithm 1 provides a sound and complete method for synthesizing a winning strategy for the controller in *G* after out-of-order delay $\delta$. $\lhd$

### Message loss

A further interesting setting that arises in practice is message loss. We will show that Algorithm 1 can also solve this problem if the number of consecutive message losses in a channel is bounded by a constant $k \in {\mathbb {N}}$ and that messages actually delivered are delayed by at most 2*k* time units. We will first demonstrate the principle on message losses in the downstream channel from the environment to the controller and will later explain how it could vice versa be applied to message losses in the upstream channel. If up to *k* consecutive losses can occur in the downstream channel and if messages can further be delayed by at most 2*k* then we always have some state information at our disposal which is no older than 2*k* time units, yet state information for intermediate steps may be missing.

**Fig. 5:**
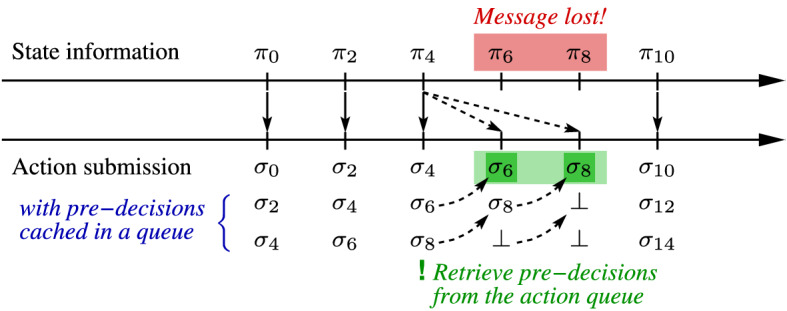
An illustration of how to win a safety game in the presence of message loss leveraging delay-resilient strategies. In the simple setting depicted here, messages cannot be delayed, but up to two consecutive messages can get lost. The controller compensates for these potential losses by at any time *t* when fresh state information is received, pre-computing two delay-resilient actions $\sigma _{t+2}$ and $\sigma _{t+4}$ in addition to the immediate action $\sigma _t$. These delayed actions become activated at their scheduled times whenever no fresh state information has arrived in the meantime

Fig. 5 depicts the central idea behind an application of Algorithm 1 for coping with that situation: at each of its turns, the controller owns some state information that has an age $t=2k'$ with $k' \le k$. If there is a strategy $(\alpha _t,\xi _t)$ that is resilient against delay $t=2k'$ then this strategy can be leveraged for computing an adequate control action despite current state information not being available. The only issue that we have to resolve in order to be able to switch forth and back between the delay-free strategy $(\varepsilon ,\xi _0)$ and different delayed strategies $(\alpha _2,\xi _2)$ to $(\alpha _{2k},\xi _{2k})$ is that all these strategies have to be compatible in that the strategies pertinent to smaller delays, though in general being more permissive, do always only visit states that are controllable under maximum delay 2*k*. This can, however, be achieved easily: as Algorithm 1 generates maximally permissive strategies and as the states from which a winning strategy originates under delay $2(m+n)$ are a subset of those winnable under delay 2*m* due to Lemma 1 (which w.l.o.g. expresses this property with respect to initial states), the maximally permissive strategies for smaller delays can be specialized such that they only traverse states winnable under the maximum delay 2*k*.

This construction justifies the following proposition:

#### Proposition 2

For a two-player safety game, there exists a winning strategy that wins when at any time $t=2n$, i.e., any player-0 move, information on the game state at some time $t'\in \{t-2k,\ldots ,t\}$ is available, iff there exists a winning strategy under an exact delay of 2*k* as formalized in Definition 5. $\lhd$

#### Proof

The “only if” part is trivial, as in-order delivery of messages with an exact delay of 2*k* is a special case of at any time $t=2n$ possessing information on the game state at some time $t'\in \{t-2k,\ldots ,t\}$. The winning strategy for the latter case thus has to win the former also. The “if” direction is covered by the above construction. $\square$

At any time $t=2n$ possessing information on the game state at some time $t'\in \{t-2k,\ldots ,t\}$ in turn is equivalent to having a lossy downstream channel from the environment to the controller where the number of consecutive message losses in a channel is bounded by a constant $k \in {\mathbb {N}}$ and that messages actually delivered are delayed by at most 2*k* time units. The construction can be extended to losses and delays in the upstream channel to the environment by in each time unit sending (or rather trying to send, as the channel is lossy) *all* the precomputed actions $\sigma _t$ to $\sigma _{t+2k}$ and have the environment buffer them. By the condition on the loss sequence, the environment will for any time $t=2n$, i.e., for any player-0 move, have a pertinent control action at its disposal.

Now, we are ready to present the main result of this section, which claims the equivalence of qualitative controllability:

#### Theorem 4

(Equivalence) Given a two-player safety game, the following statements are equivalent if $\delta$ is even:

- There exists a winning strategy under an exact delay of $\delta$, i.e., if at any point of time *t* the control strategy is computed based on a prefix of the game that has length $t-\delta$.
- There exists a winning strategy under time-stamped out-of-order delivery with a maximum delay of $\delta$, i.e., if at any point of time *t* the control strategy is computed based on the complete prefix of the game of length $t-\delta$ plus potentially available partial knowledge of the game states between $t-\delta$ and *t*.
- There exists a winning strategy when at any time $t=2n$, i.e., any player-0 move, information on the game state at some time $t'\in \{t-2k,\ldots ,t\}$ is available, i.e., under out-of-order delivery of messages with a maximum delay of $\delta$ and a maximum number of consecutively lost upstream or downstream messages of $\frac{\delta }{2}$.

The first two equivalences do also hold for odd $\delta$. $\lhd$

#### Proof

The statement follows immediately from Propositions 1 and  2. $\square$

## Case study and experimental evaluation

Avoiding collisions is a central issue in transportation systems as well as in many other applications. The task of a collision avoidance (CA) system is to track objects of potential collision risk and determine any action to avoid or mitigate a collision. One of the challenges in designing a CA system is to determine the correct action in presence of the end-to-end latency of the overall control system.

In the context of avoiding collisions, we present in this section an escape game as an artificial scenario to illustrate our synthesis approach to handling fixed delays, out-of-order delivery and bounded message loss, followed by a performance comparison of our prototypical implementation in Mathematica^1^ of Algorithm 1 against existing reduction-based methods and tools.

### Robot escape games under delays

**Fig. 6:**
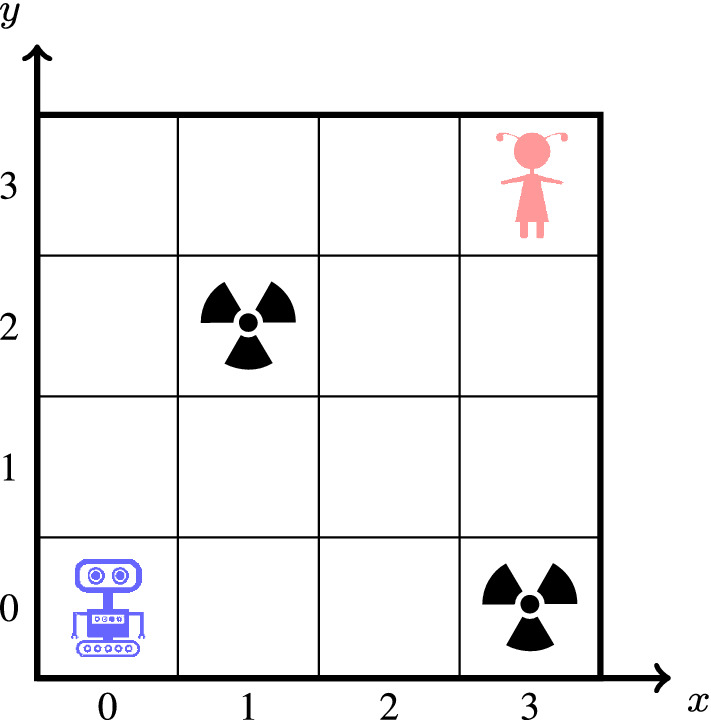
The robot escape game

The game we consider is a two-player game between a robot (i.e., the controller) and a kid (i.e., the dynamical part of its environment), which are moving in a closed room with some fixed obstacles as shown in Fig. 6. In this scenario, the robot has to make decisions (a.k.a. actions) under $\delta$-*delayed information*.

#### Definition 6

*(Two-player escape game in a* $p \times q$ *room under delay)* A *two-player escape game under delay* $\delta$ is of the form ${\widehat{G}}= \langle S, s_0, S_0, S_1, {\mathcal {O}}, \varSigma , {\mathcal {U}}, \rightarrow \rangle$, where

- $S = X \times Y \times X \times Y \times {\mathbb {B}}$ is a non-empty set of states providing $x \in X = \{0,\ldots ,p-1\}$ and $y \in Y = \{0,\ldots ,q-1\}$ coordinates for the robot as well as for the kid, together with a flag denoting whose move is next. Concretely, a state $(x_0,y_0,x_1,y_1,b)$ encodes that the robot currently is at position $(x_0,y_0)$, while the kid is at $(x_1,y_1)$, and that the next move is the robot’s iff *b* holds. Here $p, q \in {\mathbb {N}}_{\ge 1}$ denote the width and length of the room.
- ${\mathcal {O}} \subseteq X \times Y$ is a finite set of positions occupied by fixed obstacles.
- $\varSigma$ is a finite alphabet of *actions* for player 0 (i.e., the robot), which consists of kinematically constrained moves explained below.
- ${\mathcal {U}} \subseteq S$ is the finite set of undesirable states, which are characterized by featuring collisions with the obstacles or the kid.
- $\rightarrow \subseteq S \times (\varSigma \cup \{u\}) \times S$ is a set of labelled transitions, and
- $\delta$ is the delay in information retrieval s.t. the robot has to react on $\delta$ old information. $\lhd$

**Fig. 7:**
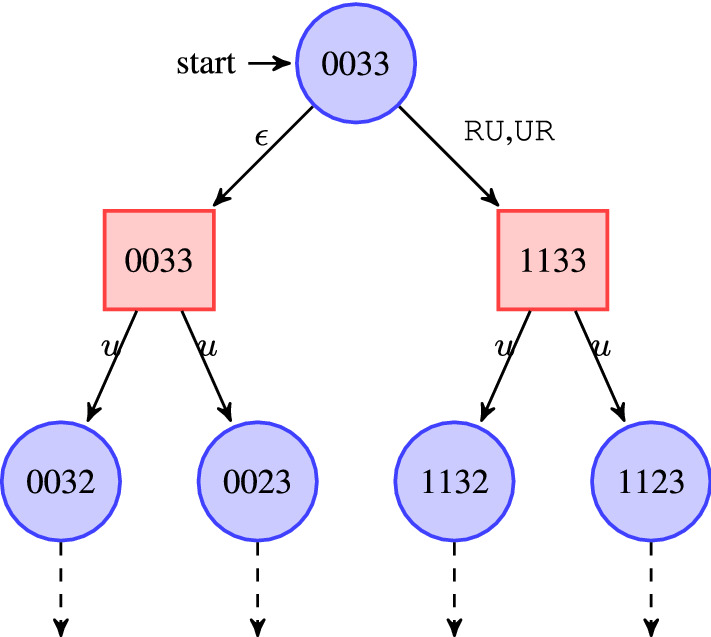
A snippet of the game graph

In our scenario, we first consider a room of extent $4 \times 4$, as shown in Fig. 6. The fixed obstacles are located at $o_1 = (1,2)$ and $o_2 = (3,0)$ and the initial state $s_0$ where the robot and the kid are located in the room is $s_0 = (0,0,3,3,\mathrm{true}) \in S_0$. The kid can move in the room and her possible moves (i.e., the *uncontrollable actions*) are unilaterally denoted by *u* for unpredictable, yet amount to moves either one step to the right ${\texttt {R}}$, left ${\texttt {L}}$, up ${\texttt {U}}$, or down ${\texttt {D}}$. The robot has a finite set of moves (i.e., *controllable actions*), which are kinematically constrained as being a combination of two moves, e.g., up then right ${\texttt {UR}}$, denoted as $\varSigma = \{{\texttt {RU}}, {\texttt {UR}}, {\texttt {LU}}, {\texttt {UL}}, {\texttt {RD}}, {\texttt {DR}}, {\texttt {LD}}, {\texttt {DL}}, \epsilon \}$, and $\epsilon$ means doing nothing. We assume that the two players respect the geometry of the room and consequently never take any action leaving the inside area of the room or running through an obstacle. Hence, each action taken by the players should satisfy certain conditions to meet these assumptions. For instance, when the robot takes ${\texttt {UR}}$ action that leads a play from $(x_0,y_0,x_1,y_1, \mathrm{true})$ to $(x'_0,y'_0,x'_1,y'_1, \mathrm{false})$, this action should satisfy the constraint ${\mathcal {C}}$, which consists of the following four conditions:

- ${\mathcal {C}}_1 := (x'_0 - x_0 = 1) \wedge (y'_0 - y_0 = 1) \wedge (x'_1 = x_1) \wedge (y'_1 = y_1)$, which describes how the robot moves.
- ${\mathcal {C}}_2 := \lnot (y_1 - y_0 = 1 \wedge x_0 = x_1)$, which prohibits the robot from running into the kid, namely the collision with the kid occurs in the first direction of its move.
- ${\mathcal {C}}_3 := (x_0, y_{0} +1) \not \in {\mathcal {O}}$, which prohibits the robot from running through an obstacle during the first direction of its move (that the endpoint of the move is outside obstacles is taken care off by the safety condition).
- ${\mathcal {C}}_4 := \forall x_0,y_0,x'_0,y'_0 :0 \le x_0,y_0,x'_0,y'_0 \le 3$, which restricts the robot to move inside the room area and avoid running into the walls.

The four conditions are instantiated for each available action and the robot must during the game only choose from legal actions satisfying the corresponding constraint ${\mathcal {C}}$. An analogous constraint system ${\mathcal {E}}$ defines the possible actions of the kid.

The safety objective for the robot is to move inside the working room while avoiding to ever be collocated with the kid or the fixed obstacles. We consequently define the set of unsafe states as

$$\begin{aligned} {\mathcal {U}}=\{(x_0,y_0,x_1,y_1,b) \mid (x_0,y_0) \in {\mathcal {O}} \vee (x_0,y_0)=(x_1,y_1)\}. \end{aligned}$$

For simplicity encoding a state $(x_0,y_0,x_1,y_1,b)$ by $x_0 y_0 x_1 y_1$ inside a blue circular node if *b* and inside a red square node if $\lnot b$, we can depict the game graph as shown in Fig. 7. The states where the robot takes actions selected from $\varSigma$ are thus circles in blue, and the states where the kid takes uncontrollable actions are the rectangles in red. From the initial state $s_0$, where the robot and the kid are located in the room, the robot in this example has only two moving actions from $\varSigma$ (i.e., RU and UR), with the other options being blocked by contact to obstacles or the walls of the room, and do nothing action $\epsilon$. Suppose that the robot will choose from its legal moving action, on both actions, the robot ends up in position (1, 1), leading to state $s_1 = (1,1,3,3,\mathrm{false})$, where it is the kid’s turn to move. The kid in this example now has only two uncontrollable actions from *u*, namely a left move ${\texttt {L}}$ and a down move ${\texttt {D}}$. The uncontrollable actions lead us to two possible successor states $s_2 = (1,1,2,3,\mathrm{true})$ or $s_3 = (1,1,3,2,\mathrm{true})$ .

### Syntheses of delay-resilient control

*Under constant delays.* There obviously exists a winning strategy for the robot in a delay-free setting, namely to cycle around the obstacle at $o_1$ to avoid being caught by the kid. To investigate the controllability resilient to fixed delays, we first construct the graph structure from the symbolic description by a C++ program. It consists of 224 states, 16 unsafe states, and 738 legal transitions satisfying the respective conditions ${\mathcal {C}}$ and ${\mathcal {E}}$. The obtained game graph is then used as input to our implementation of Algorithm 1, which declares ${\texttt {WINNING}}$ paired with a finite-memory winning strategy (i.e., a safe controller) ${\hat{\xi }}_\delta$ under delays $0 \le \delta \le 2$ (see Appendix A), while ${\texttt {LOSING}}$ when the delay is 3. The latter indicates that the problem is uncontrollable under any delay $\delta ' \ge 3$.

*With out-of-order delivery and bounded message loss.* Strictly sequential delay may likely not be guaranteed in the communication networks underlying practical transportation systems, where, e.g., the wireless vehicle-to-vehicle communication is subject to collisions and thereby prone to out-of-order delivery due to retries or even lost messages due to exceeding the maximum number of retries. These issues, however, can be tackled by the qualitative equivalence result established in Theorem 4, thereby exploiting the very same underlying strategy as the in-order case: the delay-resilient strategies (cf. Appendix A) derived under incremental delays can be utilized to assemble winning strategies for the latter cases, following the constructive proofs to Propositions 1 and 2. We therefore in the sequel do not provide any independent measurements for runtimes of strategy synthesis for the cases of out-of-order delivery or bounded message loss, as these coincide with the runtimes for the in-order case.

### Evaluation of performance

To further investigate the scalability and efficiency of our method, we have evaluated the implementation on evasion games instantiated to rooms of different sizes (marked with prefix Escp.) as well as two additional examples (Examples 3 and 4):

#### Example 3

**Fig. 8:**
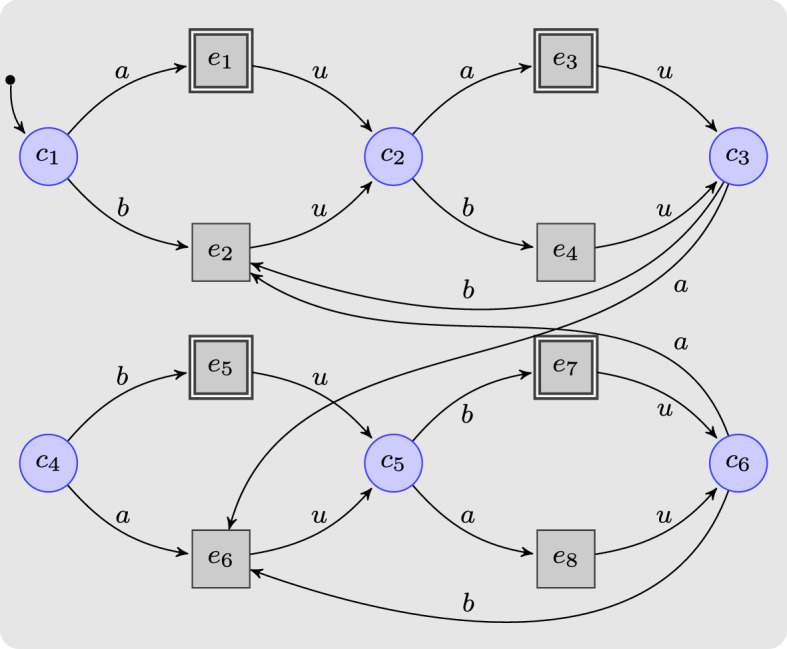
A safety game winnable for the controller with finite-memory strategies with $\delta _{\max } \ge 30$

Consider the safety game $G = \langle S, s_0, S_0, S_1, \varSigma , {\mathcal {U}}, \rightarrow \rangle$, illustrated in Fig. 8, where $S = S_0 \cup S_1$, with $S_0 = \{c_1, c_2, c_3, c_4, c_5, c_6\}$, and $S_1 = \{e_1, e_2, e_3, e_4, e_5, e_6, e_7, e_8\}$, while $s_0 = c_1$, $\varSigma = \{a, b\}$, and ${\mathcal {U}}= \{e_1, e_3, e_5, e_7\}$. $\lhd$

#### Example 4

The game in this example shares the same graph structure as that in Fig. 8, except that we empower the environment there a bit by introducing two fresh transitions $e_4 \xrightarrow {u} c_2$ and $e_4 \xrightarrow {u} c_6$ in *G*. The winning strategy then vanishes when the delay is lifted to 3. $\lhd$

A slightly adapted scenario (denoted by prefix Stub.) was also investigated, where the kid plays in a rather stubborn way, namely she always moves either one step to the left or down, yet never goes right nor up, which yields potentially larger affordable delays for the robot. In particular, a comparison of the performance of our incremental algorithm was done with respect to two points of reference: to the same Mathematica-based algorithm using $\delta =0$ (the underlying explicit-state delay-free safety synthesis) employed after reducing the games to delay-free ones by shift registers (cf. Lemma 2), and to the state-of-the-art synthesizer SafetySynth^2^ for solving safety games applied to an appropriate symbolic form of that shift-register reduction. All experiments were pursued on a 2.5 GHz Intel Core-i7 processor with 8 GB RAM running 64-bit Ubuntu 17.04.

**Table 1:** Benchmark results in relation to reduction-based approaches (time in seconds)

Benchmark				Reduction + explicit-state synthesis						Algorithm 1						
Name	|*S*|	$|\!\rightarrow \!|$	$|{\mathcal {U}}|$	$\delta _{\max }$	$\delta =0$	$\delta =1$	$\delta =2$	$\delta =3$	$\delta =4$	$\delta _{\max }$	$\delta =0$	$\delta =1$	$\delta =2$	$\delta =3$	$\delta =4$	$\%$
Example 3	14	20	4	$\ge 22$	0.00	0.00	0.01	0.02	0.02	$\varvec{\ge 30}$	0.00	0.00	**0.00**	**0.01**	**0.01**	
Example 4	14	22	4	$=2$	0.00	0.01	0.01	0.02	–	$=2$	0.00	**0.00**	**0.00**	**0.01**	–	81.97
Escp.${\texttt {4}} \times {\texttt {4}}$	224	738	16	$=2$	0.08	11.66	11.73	1059.23	–	$=2$	0.08	**0.13**	**0.22**	**0.25**	–	99.02
Escp.${\texttt {4}} \times {\texttt {5}}$	360	1326	20	$= 2$	0.18	34.09	33.80	3084.58	–	$=2$	0.18	**0.27**	**0.46**	**0.63**	–	99.02
Escp.${\texttt {5}} \times {\texttt {5}}$	598	2301	26	$\ge 2$	0.46	96.24	97.10	?	?	$\varvec{=2}$	0.46	**0.68**	**1.16**	**1.71**	–	98.98
Escp.${\texttt {5}} \times {\texttt {6}}$	840	3516	30	$\ge 2$	1.01	217.63	216.83	?	?	$\varvec{=2}$	**1.00**	**1.42**	**2.40**	**4.30**	–	99.00
Escp.${\texttt {6}} \times {\texttt {6}}$	1224	5424	36	$\ge 2$	2.13	516.92	511.41	?	?	$\varvec{=2}$	**2.06**	**2.90**	**5.12**	**10.30**	–	98.97
Escp.${\texttt {7}} \times {\texttt {7}}$	2350	11097	50	$\ge 2$	7.81	2167.86	2183.01	?	?	$\varvec{=2}$	**7.71**	**10.67**	**19.04**	**52.47**	–	98.99
Escp.${\texttt {7}} \times {\texttt {8}}$	3024	14820	56	$\ge 0$	**13.07**	?	?	?	?	$\varvec{=2}$	13.44	**18.25**	**32.69**	**108.60**	–	99.01

Benchmark		Reduction + Yosys + SafetySynth$^4$ (symbolic)							Algorithm 1 (simple explicit-state implementation)							
Name	$\delta _{\max }$	$\delta =0$	$\delta =1$	$\delta =2$	$\delta =3$	$\delta =4$	$\delta =5$	$\delta =6$	$\delta =0$	$\delta =1$	$\delta =2$	$\delta =3$	$\delta =4$	$\delta =5$	$\delta =6$	$\%$
Stub.${\texttt {4}} \times {\texttt {4}}$	$=2$	1.07	1.24	1.24	1.80	–	–	–	**0.04**	**0.07**	**0.12**	**0.18**	–	–	–	98.98
Stub.${\texttt {4}} \times {\texttt {5}}$	$=2$	1.16	1.49	1.49	2.83	–	–	–	**0.08**	**0.14**	**0.25**	**0.44**	–	–	–	98.97
Stub.${\texttt {5}} \times {\texttt {5}}$	$=2$	1.19	2.61	2.50	13.67	–	–	–	**0.21**	**0.37**	**0.63**	**1.17**	–	–	–	98.97
Stub.${\texttt {5}} \times {\texttt {6}}$	$=2$	1.18	2.60	2.59	23.30	–	–	–	**0.42**	**0.69**	**1.20**	**2.49**	–	–	–	98.96
Stub.${\texttt {6}} \times {\texttt {6}}$	$=4$	1.17	2.76	2.74	19.96	19.69	655.24	–	**0.93**	**1.47**	**2.60**	**5.79**	**7.54**	**7.60**	–	99.89
Stub.${\texttt {7}} \times {\texttt {7}}$	$=4$	**1.23**	**2.50**	**2.48**	24.57	**23.01**	2224.62	–	3.60	5.52	10.08	**22.75**	31.18	**32.98**	–	99.88

Boldface indicate better performance in terms of efficiency

$\delta _{\max }$: the maximum delay under which $G_\delta$ is controllable

$\delta _{\max } = \delta '$: $G_\delta$ is verified controllable under delays $0 \le \delta \le \delta '$ while uncontrollable under any delay $\delta > \delta '$

$\delta _{\max } \ge \delta '$: $G_\delta$ is verified controllable under delays $0 \le \delta \le \delta '$ within 1 h CPU time bound, yet unknown under $\delta > \delta '$ due to the limitation of computing capability

–: already for smaller $\delta$ the controller has no winning strategy

?: algorithm fails to answer the control/synthesis problem within 1 h of CPU time

$\%$: percentage of savings in state space compared to the reduction-based methods, as obtained on $\delta _{\max }+1$

From the upper part of Table 1, it can be seen that our incremental algorithm significantly outperforms the use of the shift-register reduction. On all cases involving delay, Algorithm 1 is faster than the same underlying explicit-state implementation of safety synthesis employed to the reduction of Lemma 2. Algorithm 1 furthermore consistently succeeds in producing an exact bound $\delta _{\max }$ on the controllable delay by proving the game uncontrollable under $\delta =\delta _{\max } + 1$, except for Example 3, where it still goes 8 steps of delay further than the reduction-based approach. In particular, the result $\delta _{\max } \ge 30$ suffices to tell that Conjecture 1, if proved, implies that the game in Example 3 is controllable under arbitrarily large delay. The benefits from not resorting to an explicit reduction, instead taking advantage of incrementally generated strategies and on-the-fly pruning of already-uncontrollable branches, are thus obvious. In contrast, the reduction-based approach suffers inevitably from the state-explosion problem: for e.g. Escp.${\texttt {4}} \times {\texttt {5}}$ under $\delta = 3$, the reduction yields a game graph comprising 29242 states and 107568 transitions.

Within the lower part of Table 1, the performance of the current explicit-state implementation of Algorithm 1 is compared with that of SafetySynth, the winner in the AIGER/safety Synthesis Track, sequential mode, of the Reactive Synthesis Competition^3^ (SYNTCOMP 2016–2018). In order to be able to examine the efficiency of our incremental algorithm under larger delays, we used a slight modification of the escape game forbidding the kid to take moves to the right or up, thus increasing the controllability for the robot. Note that Algorithm 1 completes synthesis faster in these “stubborn” scenarios due to the reduced action set. SafetySynth implements a symbolic backward fixed-point algorithm for solving delay-free safety games using the CUDD package. Its input is an extension of the AIGER^4^ format known from hardware model-checking and synthesis. We therefore provided symbolic models of the escape games in Verilog^5^ and compiled them to AIGER format using Yosys.^6^ Verilog supports compact symbolic modelling of the coordinates other than an explicit representation of the game graph as in Fig. 7, and further admits direct use of shift registers for memorizing actions of the robot under delays. Therefore, as visible in Table 1, SafetySynth outperforms our explicit-state safety synthesis for some large room sizes under small delays. For larger delays it is, however, evident that our incremental algorithm always wins, despite its use of non-symbolic encodings.

#### Remark 2

It would be desirable to pursue a comparison on standard benchmarks like the synthesis track of SYNTCOMP. As these are conveyed either in the AIGER format and not designed for modifiability, like the introduction of shift registers ; or in the TLSF/LTL format [18] where the introduction of delays may need an appropriate extension of LTL (e.g., MTL) with time constraints, this unfortunately is not yet possible. Likewise, other state-of-the-art synthesizers from the SYNTCOMP community, like AbsSynthe [6], could not be used for comparison as they do not support the state initializations appearing in the AIGER translations of the escape game. $\lhd$

## Conclusions

Designing controllers that work safely and reliably when exposed to delays is a crucial challenge in many application domains, like transportation systems or industrial robots. In this paper, we have used a straightforward, yet exponential reduction to show that the existence of a finite-memory winning strategy for the controller in games with delays is decidable with respect to safety objectives. As such a reduction being exponential in the magnitude of the delay would rapidly become unwieldy, we proposed an algorithm that incrementally synthesizes a series of controllers withstanding increasingly larger delays, thereby interleaving the unavoidable introduction of memory with state-space pruning removing all states no longer controllable under the given delay before proceeding to the next larger delay. We furthermore address the practically relevant cases of non-order-preserving delays and bounded message loss, as arising in actual networked control. To the best of our knowledge, we also provided the first implementation of such a state-space pruning within an algorithm for solving games with delays, and we demonstrated the beneficial effects of this incremental approach on a number of benchmarks.

The benchmarks used were robot escape games indicative of collision avoidance scenarios in, e.g., traffic maneuvers. Control under delay here involves selecting appropriate safe actions or movements without yet knowing the most recent positions of the other traffic participants. Experimental results on such escape games demonstrate that our incremental algorithm outperforms reduction-based safety synthesis, irrespective of whether this safety synthesis employs naïve explicit-state or state-of-the-art symbolic synthesis methods, as available in the SafetySynth tool.

An extension to hybrid control, dealing with infinite-state game graphs described by hybrid safety games, is currently under development and will be exposed in future work, as will be extensions of the finite-state case to liveness properties by means of addressing more general acceptance conditions like Büchi acceptance. We are also moving forward to a more efficient implementation of Algorithm 1 based on symbolic encodings, like BDDs [28] or SAT [5]. A further subject of future investigation is stochastic models of out-of-order delivery of messages. As these result in a high likelihood of state information being available before the maximum transportation delay, such models can quantitatively guarantee better controllability than the worst-case scenario of always delivering messages with maximum delay addressed in this paper. We will therefore attack synthesis towards quantitative safety targets in such stochastic settings and may also exploit constructive means of manipulating probability distributions of message delays, like QoS control, within the synthesis.

## Figures and Tables

**Fig. 9:**
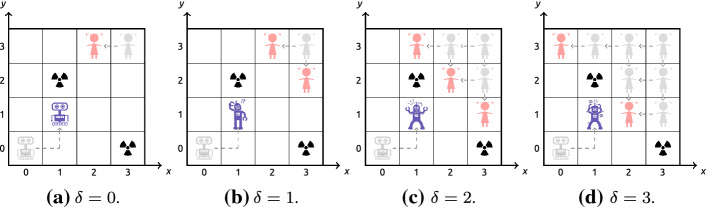
Different observations of the robot in Escp.${\texttt {4}} \times {\texttt {4}}$ when he has to make a decision at (1, 1) under different delays, with $\delta$ ranging from 0 to 3

## Appendix A Finite-memory winning strategies in Escp.${\texttt {4}} \times {\texttt {4}}$

Relabelling states: r for robot, k for kid.

Relabelling actions: $$\{{\texttt {a}}:{\texttt {UL}},{\texttt {b}}:{\texttt {LU}},{\texttt {c}}:{\texttt {UR}},{\texttt {d}}:{\texttt {RU}},{\texttt {e}}:{\texttt {DL}},{\texttt {f}}:{\texttt {LD}},{\texttt {g}}:{\texttt {DR}},{\texttt {h}}:{\texttt {RD}},{\texttt {i}}:\epsilon \}$$.

Fig. 9 depicts snapshots of the game Escp.${\texttt {4}} \times {\texttt {4}}$ under different delays and the resulting different observations of the robot. The intuition tells that

- Under $\delta = 0$, the robot always wins by cycling around $o_1 = (1, 2)$, which is encoded in the following generated maximally permissive winning strategy (see, e.g., the colored components):
- Under $\delta = 1$, the robot wins by 1-step pre-decision, with the generated winning strategy as
  $$\begin{aligned} {\hat{\xi }}_1= & {} \{ (\text {k1133},\varepsilon )\mapsto \{{\texttt {b}},{\texttt {e}},{\texttt {f}},{\texttt {g}},{\texttt {h}},{\texttt {i}}\},(\text {k0033},\varepsilon )\mapsto \{{\texttt {c}},{\texttt {d}},{\texttt {i}}\},(\text {k0223},\varepsilon )\mapsto \{{\texttt {g}},{\texttt {i}}\},\\&(\text {k2223},\varepsilon )\mapsto \{\},(\text {k0023},\varepsilon )\mapsto \{{\texttt {c}},{\texttt {d}}\},(\text {k2023},\varepsilon )\mapsto \{{\texttt {a}},{\texttt {b}}\},(\text {k1123},\varepsilon )\mapsto \{{\texttt {b}},{\texttt {i}}\},\\&(\text {k0232},\varepsilon )\mapsto \{{\texttt {c}},{\texttt {g}},{\texttt {i}}\},(\text {k2232},\varepsilon )\mapsto \{\},(\text {k0032},\varepsilon )\mapsto \{{\texttt {c}},{\texttt {d}}\},(\text {k2032},\varepsilon )\mapsto \{{\texttt {a}},{\texttt {b}}\},\\&(\text {k1132},\varepsilon )\mapsto \{{\texttt {b}},{\texttt {i}}\},(\text {k1313},\varepsilon )\mapsto \{\},(\text {k1113},\varepsilon )\mapsto \{{\texttt {e}},{\texttt {f}},{\texttt {g}},{\texttt {h}},{\texttt {i}}\},(\text {k0213},\varepsilon )\mapsto \{{\texttt {g}}\},\\&(\text {k1333},\varepsilon )\mapsto \{{\texttt {f}}\},(\text {k0233},\varepsilon )\mapsto \{{\texttt {g}},{\texttt {i}}\},(\text {k1322},\varepsilon )\mapsto \{{\texttt {f}}\},(\text {k1122},\varepsilon )\mapsto \{{\texttt {b}}\},\\&(\text {k0222},\varepsilon )\mapsto \{{\texttt {i}}\},(\text {k3313},\varepsilon )\mapsto \{\},(\text {k3113},\varepsilon )\mapsto \{{\texttt {f}}\},(\text {k2213},\varepsilon )\mapsto \{{\texttt {e}}\},(\text {k3333},\varepsilon )\mapsto \{\},\\ \end{aligned}$$
  $$\begin{aligned}&(\text {k3133},\varepsilon )\mapsto \{{\texttt {f}}\},(\text {k2233},\varepsilon )\mapsto \{{\texttt {e}}\},(\text {k3322},\varepsilon )\mapsto \{\},(\text {k3122},\varepsilon )\mapsto \{\},(\text {k2222},\varepsilon )\mapsto \{\},\\&(\text {k0013},\varepsilon )\mapsto \{{\texttt {c}},{\texttt {d}},{\texttt {i}}\},(\text {k0022},\varepsilon )\mapsto \{\},(\text {k2013},\varepsilon )\mapsto \{{\texttt {a}},{\texttt {b}},{\texttt {i}}\},(\text {k2033},\varepsilon )\mapsto \{{\texttt {a}},{\texttt {b}},{\texttt {i}}\},\\&(\text {k2022},\varepsilon )\mapsto \{\},(\text {k1331},\varepsilon )\mapsto \{{\texttt {f}},{\texttt {i}}\},(\text {k1131},\varepsilon )\mapsto \{{\texttt {b}}\},(\text {k0231},\varepsilon )\mapsto \{{\texttt {c}},{\texttt {i}}\},\\&(\text {k3331},\varepsilon )\mapsto \{\},(\text {k3131},\varepsilon )\mapsto \{\},(\text {k2231},\varepsilon )\mapsto \{{\texttt {a}}\},(\text {k0031},\varepsilon )\mapsto \{\},(\text {k2031},\varepsilon )\mapsto \{\},\\&(\text {k2203},\varepsilon )\mapsto \{{\texttt {e}},{\texttt {g}},{\texttt {h}},{\texttt {i}}\},(\text {k1303},\varepsilon )\mapsto \{\},(\text {k1323},\varepsilon )\mapsto \{\},(\text {k0203},\varepsilon )\mapsto \{\},\\&(\text {k0003},\varepsilon )\mapsto \{{\texttt {c}},{\texttt {d}}\},(\text {k2003},\varepsilon )\mapsto \{{\texttt {a}},{\texttt {b}},{\texttt {c}},{\texttt {i}}\},(\text {k1103},\varepsilon )\mapsto \{{\texttt {d}},{\texttt {g}},{\texttt {h}},{\texttt {i}}\},\\&(\text {k1332},\varepsilon )\mapsto \{{\texttt {f}},{\texttt {i}}\},(\text {k0221},\varepsilon )\mapsto \{{\texttt {c}},{\texttt {i}}\},(\text {k2221},\varepsilon )\mapsto \{\},(\text {k1321},\varepsilon )\mapsto \{{\texttt {f}},{\texttt {i}}\},\\&(\text {k0021},\varepsilon )\mapsto \{\},(\text {k2021},\varepsilon )\mapsto \{\},(\text {k1121},\varepsilon )\mapsto \{\},(\text {k3303},\varepsilon )\mapsto \{{\texttt {e}},{\texttt {f}}\},(\text {k3323},\varepsilon )\mapsto \{\},\\ \end{aligned}$$
  $$\begin{aligned}&(\text {k3103},\varepsilon )\mapsto \{{\texttt {a}},{\texttt {b}},{\texttt {f}},{\texttt {i}}\},(\text {k3123},\varepsilon )\mapsto \{\},(\text {k3332},\varepsilon )\mapsto \{\},(\text {k3132},\varepsilon )\mapsto \{\},\\&(\text {k3321},\varepsilon )\mapsto \{\},(\text {k3121},\varepsilon )\mapsto \{\},(\text {k1302},\varepsilon )\mapsto \{{\texttt {h}}\},(\text {k3302},\varepsilon )\mapsto \{{\texttt {e}},{\texttt {f}},{\texttt {i}}\},\\&(\text {k1102},\varepsilon )\mapsto \{{\texttt {d}}\},(\text {k3102},\varepsilon )\mapsto \{{\texttt {a}},{\texttt {b}},{\texttt {i}}\},(\text {k2202},\varepsilon )\mapsto \{{\texttt {c}},{\texttt {d}},{\texttt {g}},{\texttt {h}},{\texttt {i}}\},(\text {k0202},\varepsilon )\mapsto \{\},\\&(\text {k0002},\varepsilon )\mapsto \{\},(\text {k2002},\varepsilon )\mapsto \{{\texttt {c}}\},(\text {k1311},\varepsilon )\mapsto \{{\texttt {i}}\},(\text {k1111},\varepsilon )\mapsto \{\},(\text {k0211},\varepsilon )\mapsto \{{\texttt {c}}\},\\&(\text {k1320},\varepsilon )\mapsto \{{\texttt {f}},{\texttt {i}}\},(\text {k1120},\varepsilon )\mapsto \{{\texttt {b}}\},(\text {k0220},\varepsilon )\mapsto \{{\texttt {c}},{\texttt {i}}\},(\text {k3311},\varepsilon )\mapsto \{\},\\&(\text {k3111},\varepsilon )\mapsto \{\},(\text {k2211},\varepsilon )\mapsto \{{\texttt {a}}\},(\text {k3320},\varepsilon )\mapsto \{\},(\text {k3120},\varepsilon )\mapsto \{\},(\text {k2220},\varepsilon )\mapsto \{{\texttt {a}}\},\\ \end{aligned}$$
  $$\begin{aligned}&(\text {k0011},\varepsilon )\mapsto \{\},(\text {k0020},\varepsilon )\mapsto \{\},(\text {k2011},\varepsilon )\mapsto \{\},(\text {k2020},\varepsilon )\mapsto \{\},(\text {k0201},\varepsilon )\mapsto \{\},\\&(\text {k2201},\varepsilon )\mapsto \{{\texttt {a}},{\texttt {i}}\},(\text {k1301},\varepsilon )\mapsto \{{\texttt {h}},{\texttt {i}}\},(\text {k3301},\varepsilon )\mapsto \{{\texttt {e}},{\texttt {f}}\},(\text {k0001},\varepsilon )\mapsto \{\},\\&(\text {k2001},\varepsilon )\mapsto \{\},(\text {k1101},\varepsilon )\mapsto \{\},(\text {k3101},\varepsilon )\mapsto \{{\texttt {a}},{\texttt {b}}\},(\text {k0210},\varepsilon )\mapsto \{{\texttt {c}},{\texttt {i}}\},\\ \end{aligned}$$
  $$\begin{aligned}&(\text {k2210},\varepsilon )\mapsto \{{\texttt {a}},{\texttt {i}}\},(\text {k1310},\varepsilon )\mapsto \{{\texttt {f}},{\texttt {h}},{\texttt {i}}\},(\text {k0010},\varepsilon )\mapsto \{\},(\text {k2010},\varepsilon )\mapsto \{\},\\&(\text {k1110},\varepsilon )\mapsto \{\},(\text {k3310},\varepsilon )\mapsto \{{\texttt {e}},{\texttt {f}}\},(\text {k3110},\varepsilon )\mapsto \{{\texttt {a}},{\texttt {b}}\},(\text {k1300},\varepsilon )\mapsto \{{\texttt {h}},{\texttt {i}}\},\\&(\text {k1100},\varepsilon )\mapsto \{{\texttt {d}}\},(\text {k0200},\varepsilon )\mapsto \{{\texttt {c}}\},(\text {k3300},\varepsilon )\mapsto \{{\texttt {e}},{\texttt {f}},{\texttt {i}}\},(\text {k3100},\varepsilon )\mapsto \{{\texttt {a}},{\texttt {b}},{\texttt {i}}\},\\&(\text {k2200},\varepsilon )\mapsto \{{\texttt {a}},{\texttt {c}},{\texttt {d}},{\texttt {g}},{\texttt {h}},{\texttt {i}}\},(\text {k0000},\varepsilon )\mapsto \{\},(\text {k2000},\varepsilon )\mapsto \{{\texttt {c}}\}\ \}. \end{aligned}$$
- Under $\delta = 2$, the robot still wins, yet extra memory is needed. The corresponding winning strategy is analogous to that under $\delta = 1$, except that an action is prepended in the history sequence (which is previously $\varepsilon$). Thus we omit the detailed strategy here for the sake of brevity.
- Under $\delta = 3$, however, our synthesis algorithm reports LOSING, since the robot is no longer winnable (controllable), as can be seen immediately from Fig. 9d.

## Footnotes

1 Available at http://lcs.ios.ac.cn/~chenms/tools/DGame.tar.bz2. We opted for an implementation in Mathematica due to its built-in primitives for visualization.

2 https://www.react.uni-saarland.de/tools/safetysynth/.

3 http://www.syntcomp.org/.

4 http://fmv.jku.at/aiger/.

5 http://www.verilog.com/.

6 http://www.clifford.at/yosys/.
